# Targeting rapid TKI‐induced AXL upregulation overcomes adaptive ERK reactivation and exerts antileukemic effects in *FLT3*/ITD acute myeloid leukemia

**DOI:** 10.1002/1878-0261.13749

**Published:** 2024-10-12

**Authors:** Tessa S. Seale, Li Li, J. Kyle Bruner, Melody Chou, Bao Nguyen, Jaesung Seo, Ruiqi Zhu, Mark J. Levis, Christine A. Pratilas, Donald Small

**Affiliations:** ^1^ Department of Oncology Johns Hopkins University School of Medicine Baltimore MD USA; ^2^ Department of Pediatrics Johns Hopkins University School of Medicine Baltimore MD USA

**Keywords:** adaptive response, cancer resistance, *FLT3*/ITD AML, RTK signaling, targeted therapy

## Abstract

Acute myeloid leukemia (AML) patients with the FMS‐related receptor tyrosine kinase 3 internal tandem duplication (*FLT3*/ITD) mutation have a poorer prognosis, and treatment with FLT3 tyrosine kinase inhibitors (TKIs) has been hindered by resistance mechanisms. One such mechanism is known as adaptive resistance, in which downstream signaling pathways are reactivated after initial inhibition. Past work has shown that *FLT3*/ITD cells undergo adaptive resistance through the reactivation of extracellular signal‐regulated kinase (ERK) signaling within 24 h of sustained FLT3 inhibition. We investigated the mechanism(s) responsible for this ERK reactivation and hypothesized that targeting tyrosine‐protein kinase receptor UFO (AXL), another receptor tyrosine kinase that has been implicated in cancer resistance, may overcome the adaptive ERK reactivation. Experiments revealed that AXL is upregulated and activated in *FLT3*/ITD cell lines mere hours after commencing TKI treatment. AXL inhibition combined with FLT3 inhibition to decrease the ERK signal rebound and to exert greater anti‐leukemia effects than with either treatment alone. Finally, we observed that TKI‐induced AXL upregulation occurs in patient samples, and combined inhibition of both AXL and FLT3 increased efficacy in our *in vivo* models. Taken together, these data suggest that AXL plays a role in adaptive resistance in *FLT3*/ITD AML and that combined AXL and FLT3 inhibition might improve *FLT3*/ITD AML patient outcomes.

## Graphical abstract

Adaptive ERK reactivation hinders FLT3 tyrosine kinase inhibitor (TKI) treatment in *FLT3*/ITD acute myeloid leukemia. Here, we report that FLT3 TKI treatment rapidly induces AXL expression and upregulation that is temporally associated with the adaptive ERK reactivation. Combined AXL and FLT3 inhibition overcomes this signal rebound and exerts greater antileukemic effects than either treatment alone.

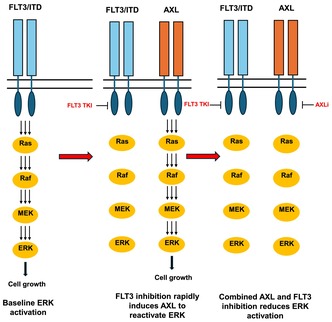

**Abbreviations**
AKT: RAC‐alpha serine/threonine‐protein kinase
AML: acute myeloid leukemia
AP‐1: activator protein‐1
ERK: extracellular signal‐regulated kinase
*FLT3*/ITD: FMS‐related receptor tyrosine kinase 3 internal tandem duplication
GAS6: growth arrest‐specific protein 6
JAK: Janus Kinase
MEK: mitogen‐activated protein kinase kinase
NSCLC: non‐small cell lung cancer
PI3K: phosphatidylinositol 3‐kinase
RAF: rapidly accelerated fibrosarcoma
RTK: receptor tyrosine kinase
SD: standard deviation
SEM: standard error of mean
shRNA: short‐hairpin RNA
siRNA: small interfering RNA
STAT5: signal transducer and activator of transcription 5
TAM: Tyro3 AXL MERTK
TKI: tyrosine kinase inhibitor
YAP: Yes‐associated protein

## 1 Introduction

FMS‐like tyrosine kinase‐3 (FLT3) is a class III receptor tyrosine kinase (RTK) that is the most frequently mutated gene in acute myeloid leukemia (AML) [1]. The internal tandem duplication (*FLT3*/ITD) is the most common FLT3 mutation, occurring in roughly 23% of adult AML patients [1, 2]. These mutations result in constitutive activation of the receptor that in turn activates downstream pathways [3, 4]. Furthermore, AML patients with the *FLT3*/ITD mutation have a poorer prognosis and overall survival [2].

The constitutive FLT3 activation makes *FLT3*‐mutated AML a validated candidate for treatment with tyrosine kinase inhibitors (TKI) [5, 6]. First‐generation and second‐generation FLT3 TKI have been shown to inhibit FLT3, and some have achieved a degree of clinical efficacy [5, 7]. However, the clinical success of these FLT3 TKI has been limited by several mechanisms of resistance. Among these are selection for additional mutations within FLT3 rendering the drugs no longer able to bind [8, 9, 10] and upregulation of FLT3 ligand [11, 12]. FLT3‐mutated relapsed and refractory patients have dismal outcomes, and while gilteritinib treatment prolongs survival compared to salvage chemotherapy, it is not curative [13]. Thus, elucidating and targeting FLT3 TKI resistance has immense clinical importance.

Past work in our lab has shown that *FLT3*/ITD AML cells treated with FLT3 TKI experience an initial inhibition of ERK signaling followed by a reactivation of ERK that starts within a few hours after commencing TKI treatment [14]. Such rapid signal reactivation in response to a targeted treatment is known as an “adaptive response” [15] or “adaptive resistance” [16, 17]. While acquired resistance involves genetic changes occurring over long‐term treatment, adaptive resistance is rapid and non‐genetic. Both adaptive ERK and protein kinase B (AKT) reactivation have been observed in a wide range of targeted therapies against many cancers, including EGFR, HER2, MEK, RAF, and PI3K inhibition in non‐small cell lung cancer (NSCLC), renal cell carcinoma, melanoma, colorectal cancer, and prostate cancer respectively [18, 19, 20, 21, 22]. Additionally, there have been therapeutic window trials that demonstrated early adaptive responses in patients just starting treatment [23, 24].

While adaptive resistance can involve broader changes in a cancer cell's transcriptional landscape like kinome reprogramming [16], we are focused on the adaptive responses that involve reactivation of initially inhibited oncogenic pathways like the MEK/ERK and PI3K/AKT [17]. These mechanisms are characterized by the relief of negative feedback loops that normally inhibit upstream RTK signaling, thereby causing the activation of alternative RTK to reactivate the downstream signal [17]. Thus, it may be advantageous to target another RTK along with FLT3 TKI to overcome the adaptive ERK reactivation.

AXL is a RTK that is involved in resistance in several cancers. AXL belongs to the TAM family, which also includes TYRO3 and MERTK. It is activated by its ligand growth arrest‐specific protein 6 (GAS6), but it can also undergo GAS6‐independent activation via ligand‐independent homodimerization and also heterodimerization with other RTK [25, 26]. Once activated, it activates downstream pathways including PI3K, JAK/STAT, and MEK/ERK [27]. In many cancers, AXL is overexpressed and aids in the proliferation, invasiveness, and migration of tumor cells [28]. It has also been implicated in selection for resistance against both chemotherapy and targeted therapies [29, 30]. In AML specifically, AXL overexpression has been observed and is associated with a poorer prognosis [31, 32]. Park et al. reported that upon treatment with FLT3 TKI for 72 h, FLT3‐ITD AML cell lines showed increased activation of AXL. Additionally, they showed that PKC412‐resistant cells have increased AXL activation compared to sensitive cells, indicating that over time, the cells become resistant with higher levels of AXL signaling [33]. However, AXL signaling has not been examined over a shorter (24 h) period of TKI treatment or whether it plays a role in pERK signaling rebound. In this report, we examine AXL expression and activation in response to short‐term inhibition of FLT3 signaling and evaluate it as a therapeutic target against the adaptive response to FLT3 inhibition.

## 2 Materials and methods

### 2.1 Cell lines and reagents

MV4;11 (RRID: CVCL_0064), HL60 (RRID: CVCL_0002), THP‐1 (RRID: CVCL_0006), K562 (RRID: CVCL_0004), U937 (RRID: CVCL_0007), and SKBR3 (RRID: CVCL_0033) cells were purchased from ATCC (Manassas, VA, USA). Molm14 cells (RRID: CVCL_7916) were purchased from the DSMZ (Braunschweig, Germany). HCC827 (RRID: CVCL_2063) cells were obtained from C. Hann (John Hopkins University). All cell lines were authenticated with short tandem repeat profiling at different loci using PCR analysis and were determined to be mycoplasma‐free. Sorafenib, quizartinib, lestaurtinib, and crenolanib were purchased from LC labs, while trametinib, lapatinib, gilteritinib, R428, TP‐0903, LY294002, Verteporfin, and VT‐107 were obtained from Selleckchem. For *in vitro* studies, drugs were dissolved in 100% DMSO and then diluted in RPMI plus 0.1% BSA to yield a 10 μm stock that was stored at −80 °C. Human AXL fusion soluble protein (AXL‐Fc) and human control‐Fc (Ctrl‐Fc) were purchased from R&D and were stored according to manufacturer's protocol.

### 2.2 Immunoblot analysis and antibodies

Cells were lysed using cell lysis buffer (Cell Signaling Technologies, Danvers, MA, USA) following the manufacturer's protocol. The resulting lysate were quantified by BCA assay (Pierce Thermo Fisher Scientific, Waltham, MA, USA) and equal amounts of lysate were separated by SDS/PAGE and transferred to a polyvinyl difluoride (PVDF) membrane (Millipore, Burlington, MA, USA). Proteins were probed using the indicated antibodies and horseradish peroxidase‐conjugated secondary antibodies, followed by ECL detection using a Chemidoc Touch Imaging System (Bio‐Rad, Hercules, CA, USA). Phosphorylated antibodies and total protein antibodies were run on separate parallel gels. FLT3 and GAS6 were from Santa Cruz Biotechnology (Dallas, TX, USA) and actin was from Sigma (Burlington, MA, USA). All other antibodies were from Cell Signaling Technologies.

### 2.3 Primers and quantitative RT‐PCR

RNA was isolated and collected using the RNeasy Mini kit (Qiagen, Hilden, Germany). It was then converted to cDNA using iScript cDNA synthesis kit (Bio‐Rad). Quantitative RT‐PCR was then completed using an iCycler iQ multicolor real‐time PCR system (Bio‐Rad). The transcript expression levels were normalized to GAPDH levels. The primer sequences used for AXL, GAS6 and GAPDH are as follows: AXL, F‐5′‐CCT ACT CTG CCA CGA TG‐3′, R‐5′‐CGC AGG AGA AAG AGG ATG TC‐3′; GAS6, F‐5′‐AGT GCG TGG AGG AGC TGT‐3′, R‐5′‐CGA AGC CTG AGT TTT TG‐3′; GAPDH, F‐5′‐GAA GGT GAA GGT CGG AGT CA‐3′, R‐5′‐AAT GAA GGG GTC ATT GAT GG‐3′.

### 2.4 Flow cytometry analysis

Flow cytometry was performed using a BD FACSCelesta machine (BD Biosciences, Franklin Lakes, NJ, USA) Apoptosis was assayed by incubating cells with Annexin V‐PE antibody and 7‐AAD (3 μL each) for 15 min. All data were analyzed by flowjo analysis software 9.3. (flow jo llc, Ashland, OR, USA).

### 2.5 Growth inhibition

For cell proliferation, cells were seeded in triplicate at a density of 2.5 × 10^4^ cells per well. Cell proliferation was measured in triplicate using the 3‐(4,5‐dimethylthiazol‐2‐yl)‐2,5‐dimethyltetrazolium bromide (MTT) assay following the manufacturer's protocol (Roche Applied Science, Penzberg, Germany). Proliferation for cells transfected with the TRIPZ shRNA constructs was analyzed using the 3‐(4,5‐dimethylthiazol‐2‐yl)‐5‐(3‐carboxymethoxyphenyl)‐2‐(4‐sulfophenyl)‐2H‐tetrazolium (MTS) assay according to manufacturer's protocol (Promega, Madison, WI, USA). Fraction of cell viability was measured using Trypan Blue exclusion assay following manufacturer's protocol (Gibco, Billings, MA, USA).

### 2.6 Mouse studies

Our study was performed in accordance with the regulations of the Animal Welfare Act and Public Health Service (PHS) policy. Our experiments were approved by our Institutional Animal Care and Use Committee (IACUC) (Protocol number: MO22M03) at JHU. Our mice were housed in individually ventilated cages (5 mice per cage) under 12‐h light/dark shifts in our institution's Animal Core Facility under the care and supervision of the Johns Hopkins University specialized animal facility personnel. Bedding was changed weekly, acidified water was continuously supplied through a nozzle at the back of the cage, and Teklad global 18% protein extruded rodent diet (Envigo) was sterilized and provided in the cages. NOD.Cg‐*Prkdc* ^*scid*^ *Il2rg* ^*tm1Wjl*^/SzJ (NSG) 6‐week‐old female mice were acquired from the Johns Hopkins Animal Core Facility. For the pharmacological combination experiment, Molm14 cells were injected into the mice via tail vein injection. After 72 h, the mice were randomized to receive vehicle, 15 mg·kg^−1^ of gilteritinib, 30 mg·kg^−1^ of TP‐0903, or the combination once daily for 5 days of the week (no dose on the weekend) for 4 weeks. For the knockdown experiment, NSG mice were injected with either shAXL‐2 Molm14 cells or non‐specific control Molm14 cells and then treated with either 15 mg·kg^−1^ of gilteritinib, 30 mg·kg^−1^ of gilteritinib, or vehicle. 500 μg·mL^−1^ of doxycycline along with 0.5% of sucrose was administered to the mice via acidified water. The drugs were administered in corn oil by oral gavage.

### 2.7 shRNA and siRNA knockdown

AXL knockdown was performed with TRIPZ lentiviral shRNA constructs from Dharmacon (Lafayette, CO, USA) (shAXL: ID: v2ths 201 787; shAXL‐2: v2ths 202 535) and a non‐silencing inducible control (ID: RHS4743). The constructs were introduced in Molm14 cells using lentiviral packaging as previously described [34]. Pooled siRNAs against YAP and a non‐silencing control was purchased from Dharmacon and transfected into MV4;11 cells using Amaxa Nucleofector II system (Lonza, Basel, Switzerland) following the manufacturer's protocol.

### 2.8 Human samples

Human AML samples and CD34+ peripheral blood stem cells (PBSCs) from healthy donors were obtained from the Johns Hopkins Hematologic Malignancies Cell Bank (protocol: J0969) upon Johns Hopkins Institutional Review Board approval with written informed consent from patients in accordance with the Declaration of Helsinki. Our experiments also conformed with the standards set by the Declaration of Helsinki and were approved by the Johns Hopkins Institutional Review Board (IRB number: NA_00028682). Patient samples were collected from October 2011 to July 2018. Mononuclear cells were separated with Ficoll centrifugation and cryopreserved with liquid nitrogen until use. AML cells were cultured in RPMI media with 10% FBS, and CD34+ PBSCs were cultured in IMDM (Gibco) with 10% FBS and either with or without cytokines (SCF, FLT3L, IL‐3, IL‐6 and GM‐CSF, all from Peprotech, Rocky Hill, NJ, USA).

### 2.9 Statistical analysis

Unpaired student *t*‐test (two‐tailed) and log‐rank test were performed using graphpad prism software (GraphPad Software, Boston, MA, USA). Combination index values and dose reduction index values were analyzed using the Chou‐Talalay method [35].

### 2.10 RTK phospho‐array

MV4;11 cells were assayed using the PathScan® RTK signaling Antibody Array (Cell Signaling Technologies), following the manufacturer's protocol. The signaling intensity of each duplicate spot was quantified and adjusted for local background with Image Lab (Bio‐Rad). The adjusted signal intensity was then normalized against the positive control signals on each array. The 48‐h array were further normalized against the untreated array.

### 2.11 Lentiviral AXL overexpression

Constitutive AXL overexpression and doxycycline‐inducible AXL expression was performed with lentiviral constructs purchased from Addgene (Watertown, MA, USA).  [36, 37] (IDs: AXL: 116714, Control:126686, inducible AXL: 124797). The constructs were introduced in Molm14 cells using lentiviral packaging as described in the knockdown studies.

### 2.12 Colony formation assay

Cells were plated at a density of 1000 cells per mL in methylcellulose (Methocult H4435; Stem Cell Technologies, Vancouver, Canada) and incubated at 37 °C. Total colony counts and/or burst‐forming unit erythroid (BFU‐E), Colony forming unit‐granulocyte/macrophage (CFU‐GM), Colony forming unit‐granulocyte (CFU‐G), and Colony forming unit‐macrophage (CFU‐M) counts were obtained on day 14.

## 3 Results

### 3.1 FLT3 TKI treatments cause rapid AXL upregulation and activation in *FLT3*/ITD AML cell lines

**Fig. 1:**
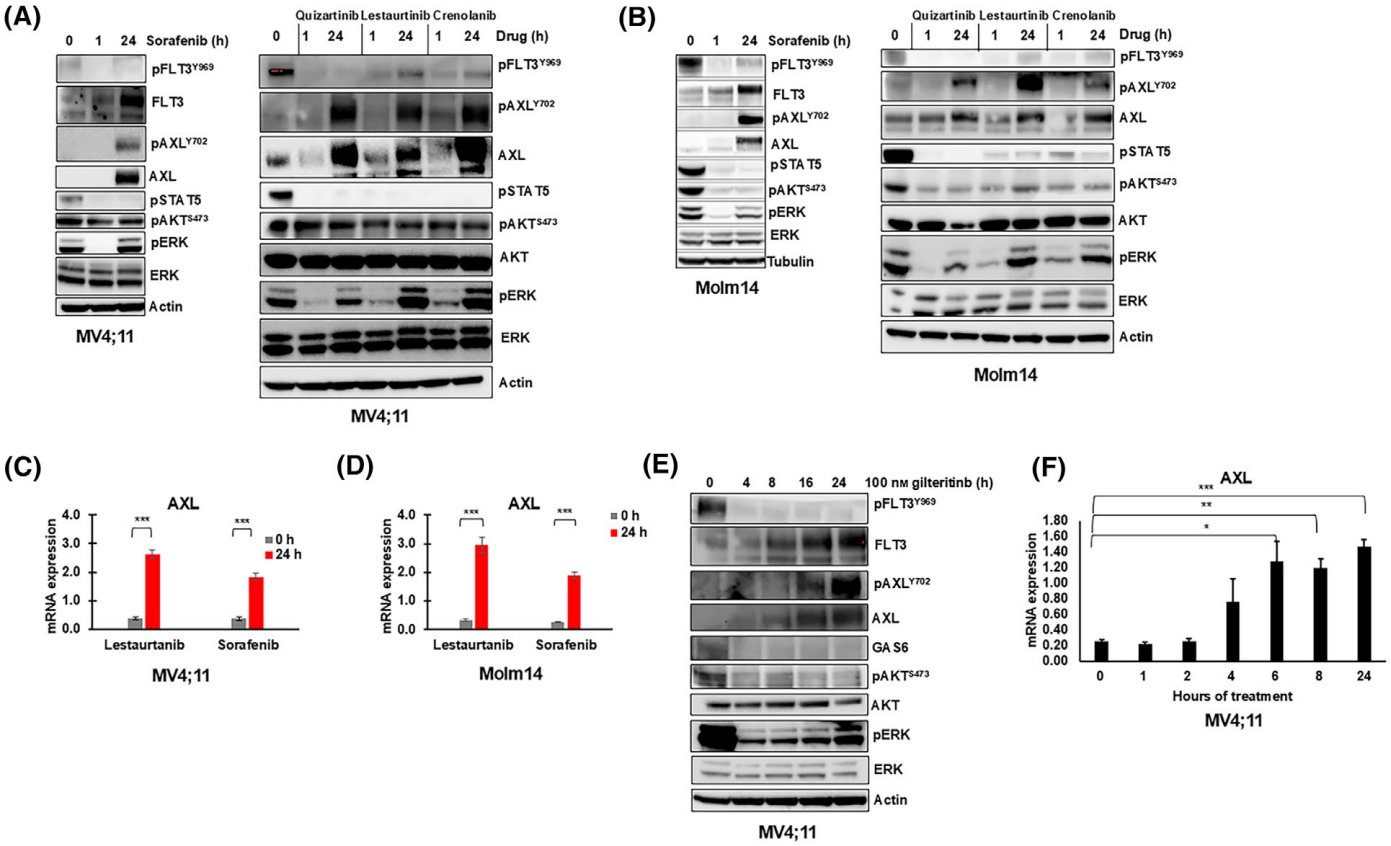
FLT3 tyrosine kinase inhibitor treatment results in AXL upregulation within hours after treatment. (A) MV4;11 and (B) Molm14 cells were treated with the indicated FLT3 tyrosine kinase inhibitors (TKI) (sorafenib: 25 nm, quizartinib: 10 nm, lestaurtinib: 25 nm, crenolanib: 25 nm), and protein lysates were collected at the indicated times. The lysates were subjected to immunoblot analysis. The blots are representative of two independent experiments. (C) MV4;11 and (D) Molm14 cells were treated with 25 nm of either sorafenib or lestaurtinib for 24 h, and AXL expression was measured in triplicate using quantitative RT‐PCR relative to glyceraldehyde 3‐phosphate dehydrogenase (GAPDH). Error bars indicate average expression ± SEM and *P* values were determined using the unpaired student *t*‐test (***P < 0.001). (E) MV4;11 cells were treated with 100 nm of gilteritinib for 24 h and protein lysate was collected at the indicated time points and subjected to immunoblot analysis. The blots are representative of two independent experiments. (F) AXL expression, measured in triplicate and determined by quantitative RT‐PCR relative to GAPDH, was analyzed at the indicated time points in MV;411 cells treated with 25 nm of sorafenib. Error bars indicate expression ± SEM and *P* values were determined using the unpaired student *t*‐test (*P < 0.05, **P < 0.01, ***P < 0.001).

Previously, we observed that sustained FLT3 inhibition using FLT3 TKI results in a rapid pERK rebound, indicating adaptive resistance. To investigate whether RTK activation may be involved, we conducted a phospho‐RTK array on the *FLT3*/ITD cell line MV4;11 treated with or without the FLT3 TKI sorafenib for 48 h. As expected, treatment of sorafenib lowered pFLT3 signal intensity (Fig. S1A,B). On the other hand, AXL was one of the few RTK with increased signal intensity after treatment. As AXL has been implicated in both long‐term resistance in AML and adaptive resistance in other cancers [30, 33], we hypothesized that targeting AXL could wholly or at least partially decrease the previously observed rapid rebound. We treated *FLT3*/ITD AML cell lines Molm14 and MV4;11 with several FLT3 TKI (sorafenib, quizartinib, lestaurtinib, crenolanib, and gilteritinib) at concentrations effective for sustained FLT3 inhibition for 24 h. We confirmed our previously reported pERK rebound and observed a strong induction of both AXL protein and AXL phosphorylation (pAXL) at 24 h in response to FLT3 TKI treatment (Fig. 1A,B,E). FLT3's other downstream targets AKT and STAT5 showed inhibition as previously observed [14]. For efficiency and to still demonstrate generalizability of responses, we used gilteritinib and sorafenib as our predominant FLT3 inhibitors in subsequent experiments. Quantitative RT‐PCR analysis revealed that AXL mRNA levels are induced at 24 h as well (Fig. 1C,D). In contrast, we observed that AXL's ligand, GAS6, showed decreased protein levels and mRNA levels in response to FLT3 TKI treatment (Fig. 1E, Fig. S1C,D). A time course experiment revealed that AXL mRNA and protein levels increase by 4 h of FLT3 TKI treatment, while increased AXL phosphorylation occurred by 16 h (Fig. 1E,F). Interestingly, this signal coincides with the reemergence of the pERK signal, demonstrating that AXL activation temporally correlates with the observed pERK rebound and might therefore play a role in its reactivation. Healthy non‐leukemic CD34+ cells and other leukemia cell lines with wild‐type FLT3 exhibited no induction of AXL protein levels when treated with FLT3 TKI, even with differing basal AXL levels across the different cells (Fig. S1E). This suggests that the AXL induction is not a non‐leukemic or general response to FLT3 inhibition and may be more specific to *FLT3*/ITD AML cells.

### 3.2 AXL upregulation is associated with ERK activation

**Fig. 2:**
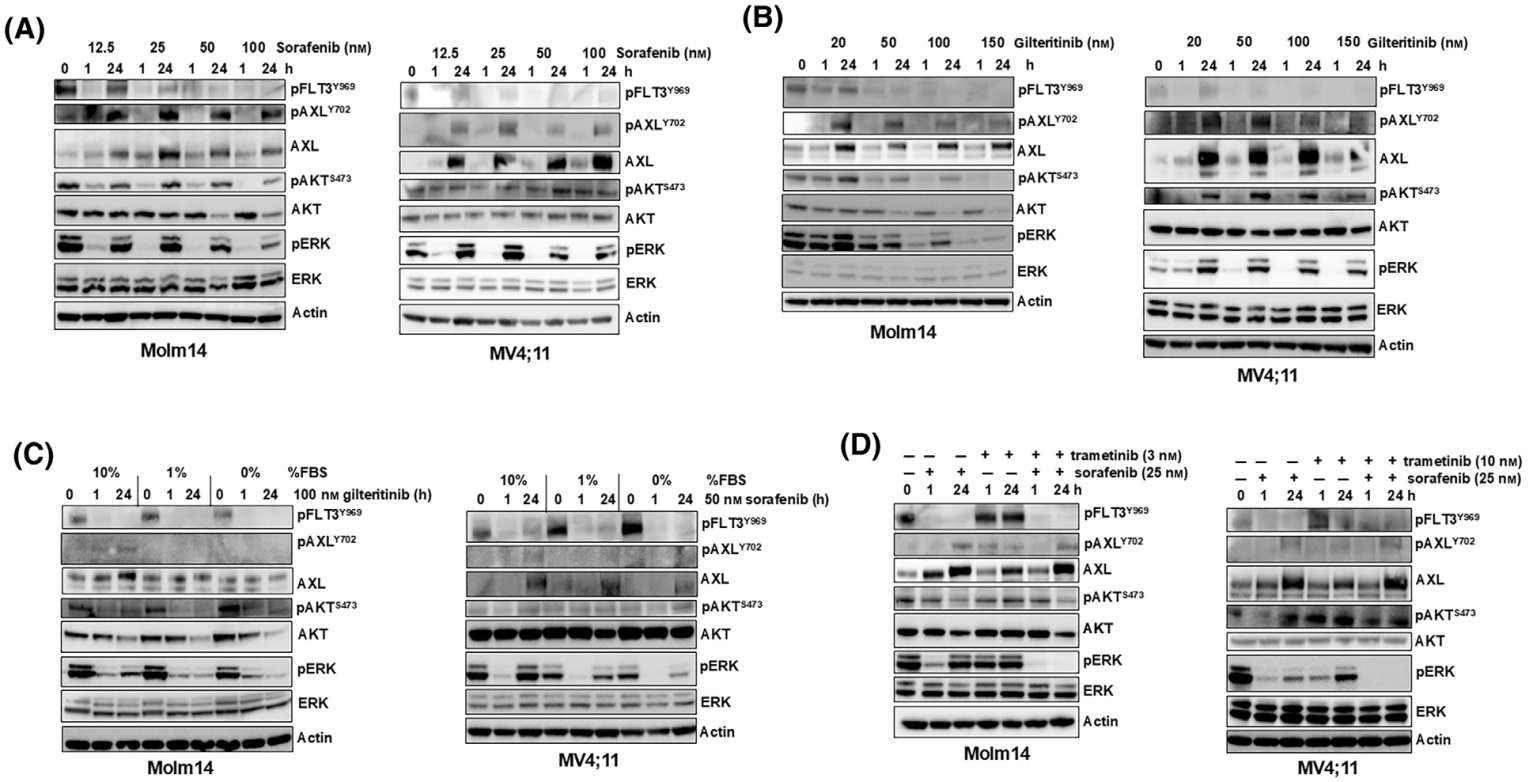
AXL signaling is associated with, but not a result of, inhibitor‐induced ERK reactivation. Molm14 and MV4;11 cells were treated with the indicated concentrations of sorafenib (A) or gilteritinib (B) for 24 h. Protein lysates were subjected to immunoblot analysis against the indicated antibodies. (C) Molm14 and MV4;11 cells were treated with the indicated FLT3 tyrosine kinase inhibitor (TKI) for 24 h in media with the indicated percent of fetal bovine serum (FBS) and protein lysates were subjected to immunoblot analysis against the indicated antibodies. (D) Molm14 and MV4;11 cells were treated with the indicated concentrations of sorafenib, trametinib, or both for 24 h. Protein lysates were subjected to immunoblot analysis. All blots are representative of two independent experiments.

We next treated these cell lines with increasing levels of FLT3 TKI to determine if there was a dose‐dependent mechanism driving AXL upregulation. Consistent with our previous observation, higher concentrations of sorafenib treatment resulted in decreased pERK rebound [14], potentially due to the inhibitory effects of sorafenib on Raf kinases as well as RTK [38]. However, levels of AXL and pAXL induction were unaffected, and the highest concentration of sorafenib (100 nm) still resulted in a pERK rebound (Fig. 2A). Since gilteritinib is a dual FLT3/AXL inhibitor, we were interested in its effects on AXL signaling. Treatment of cells with increasing concentrations of gilteritinib resulted in increased AXL protein levels but also reduced pAXL levels and pERK rebound (Fig. 2B). However, pAXL activation was not completely abrogated by higher dosages that effectively inhibited FLT3, indicating that gilteritinib is more potent against FLT3 than it is against AXL. This was observed in other studies [39, 40] and raises the possibility that AXL is activated and signaling to downstream pathways even in patients achieving effective levels of FLT3 inhibition by gilteritinib treatment.

It is notable that our dosing experiments also revealed a clear rebound effect in AKT phosphorylation (pAKT), although this was not observed as consistently or as strongly as was the pERK rebound (Fig. 2A,B). PI3K/AKT reactivation has been observed as an adaptive response in other cancers [16, 41, 42], but further investigation is required to determine if AKT reactivation is an adaptive response in *FLT3*/ITD AML. Since AXL modulates the AKT pathway, targeting AXL has the potential to reduce AKT signaling in addition to the ERK reactivation.

Our past work showed that the pERK rebound was dependent on serum [14]. Therefore, we next examined the effects of serum components on AXL upregulation. FLT3 TKI treatment of cells in the presence of decreasing levels of serum showed decreased pERK rebound, as previously observed [14], and lowered AXL induction (Fig. 2C). The fact that the level of pERK induction correlated with reduced AXL induction again implies a possible relationship between these pathways.

Up to this point, our experiments demonstrate an association between rapid AXL upregulation and adaptive ERK reactivation. To delve further into the relationship between increased AXL expression and ERK activation, we constitutively overexpressed AXL (AXL+) in Molm14 cells. After selection, we treated these cells and control cells (control) with FLT3 TKI for 4 h, since AXL expression is not heavily induced by FLT3 TKI by that time point. The AXL+ cells showed high AXL protein levels, and the pERK signal rebounded by 4 h, suggesting that overexpressed AXL creates a stronger and faster pERK rebound in response to quizartinib. Interestingly, treatment with gilteritinib lowered the pERK signal in AXL+ cells compared to control cells, likely due to AXL overexpression sensitizing cells to the AXL‐inhibiting aspect of the dual AXL/FLT3 inhibitor (Fig. S2A). To more closely recapitulate the rapid AXL induction observed with FLT3 TKI treatment, we also transduced a doxycycline‐inducible AXL construct (indAXL+) and a non‐specific control construct (indControl) into Molm14 cells. Doxycycline increased AXL expression and induced a stronger pERK rebound in indAXL+ cells, most notably at 4 and 8 h of sorafenib treatment (Fig. S2B). AXL overexpression also enhanced proliferation in both cell lines; Molm14 cells with constitutive AXL expression exhibited increased proliferation and decreased sensitivity to gilteritinib treatment when compared to control cells, and MV4;11 cells also had higher basal levels of proliferation with increased AXL expression (Fig. S2C). Taken together, these data demonstrate how AXL overexpression can induce a stronger pERK rebound signal.

Previous studies showed that AXL signaling can be regulated by the AP‐1 transcription factor family, known to be downstream of ERK signaling [28]. This relationship suggests that perhaps ERK reactivation could be the cause of the AXL overexpression, as opposed to AXL signaling causing the ERK reactivation. Therefore, we treated Molm14 and MV4;11 cell lines with either a MEK inhibitor alone, a FLT3 TKI alone, or the combination (Fig. 2D, Fig. S3A). The combination of the MEK inhibitor with FLT3 TKI abrogated the pERK rebound, as seen before in our previous work [14]. However, AXL induction at the level of phosphorylation, protein and mRNA was unchanged when compared to treatment with a FLT3 TKI alone, suggesting that the pERK rebound is not responsible for AXL upregulation and activation. Interestingly, treatment with trametinib alone also caused an increase in AXL protein and mRNA levels (Fig. 2D, Fig. S3B) suggesting that MEK inhibition, like FLT3 inhibition, results in downstream perturbations that cause AXL upregulation.

While the addition of a MEK inhibitor failed to affect AXL upregulation, the addition of the pan‐PI3K inhibitor LY294002 to FLT3 TKI did result in lowered AXL and pERK signal (Fig. S3C). There is some evidence that the PI3K pathway and LY294002 can modulate the Yes‐associated protein (YAP), a regulatory component of the Hippo signaling pathway [43]. When phosphorylated, it is inactive and remains in the cytoplasm, but upon activation it translocates to the nucleus and activates the transcription of its downstream genes, one of which is AXL [44]. Because of this, we investigated whether YAP could be involved with the observed rapid AXL protein induction. Twenty‐four hour FLT3 TKI treatment decreased YAP phosphorylation, while the combination of PI3K and FLT3 inhibition increased YAP phosphorylation, thus associating YAP inactivation with lowered AXL induction (Fig. S3C). Next, we inhibited YAP with YAP inhibitor Verteporfin, TEAD inhibitor VT‐107, and siRNA knockdown. All methods of YAP pathway inhibition combined with FLT3 TKI to decrease AXL protein levels and downstream ERK reactivation (Fig. S3D–F). Collectively, this data implicates YAP as a potential upstream activator of the observed AXL upregulation.

### 3.3 AXL inhibition decreases FLT3 TKI‐dependent pERK reactivation and sensitizes *FLT3*/ITD cell lines to FLT3 TKI treatment

**Fig. 3:**
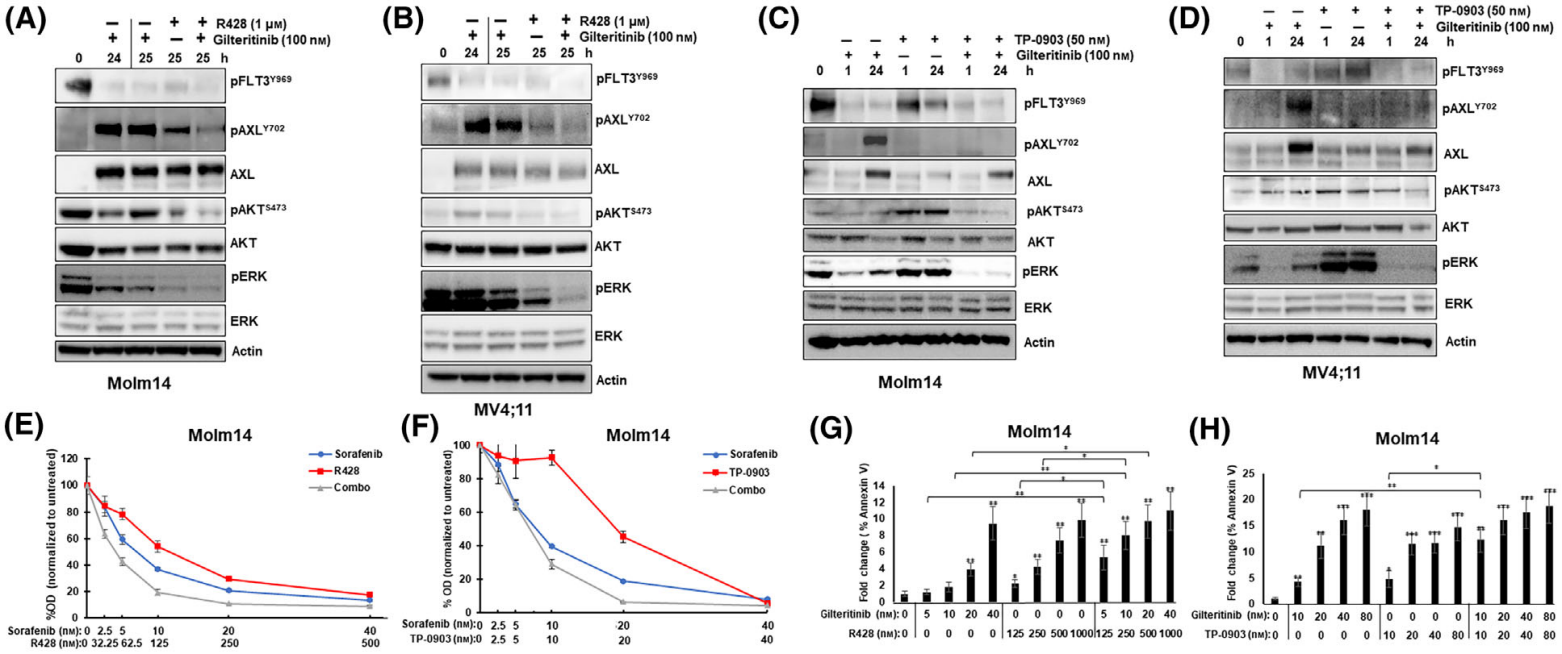
Pharmacological inhibition of AXL decreases pERK rebound and sensitizes cells to FLT3 tyrosine kinase inhibitor treatment. (A) Molm14 and (B) MV4;11 cells were treated with 100 nm of gilteritinib for 24 h and then the cells were washed free of drug and retreated with 100 nm of gilteritinib, 1 μm of R428, or the combination for an additional hour. The vertical line indicates the point of drug removal. Protein lysates were collected and analyzed by immunoblot assay. The blots are representative of two independent experiments. (C) Molm14 and (D) MV4;11 cells were treated with 100 nm of gilteritinib, 50 nm of TP‐0903, or the combination for 24 h. Protein lysates were collected and analyzed by immunoblot assay. The blots are representative of two independent experiments. (E, F) Molm14 cells were treated for 48 h with the indicated concentrations of either sorafenib alone, R428 (E) or TP‐0903 (F) alone, or the combination. Proliferation was measured by the 3‐(4,5‐dimethylthiazol‐2‐yl)‐2,5‐dimethyltetrazolium bromide (MTT) assay in triplicate. Error bars indicate average percent optical density (OD) ± SD. (G, H) Molm14 cells were treated with the Gilteritinib, R428 (G) or TP‐0903 (H) alone, or the combination for 48 h. Apoptosis was measured with Annexin V assay in biological triplicates. Error bars indicate average fold change of % Annexin V positive vs. untreated ± SD. *P* values were determined using the unpaired student *t*‐test, and asterisks directly above the error bar indicate *P*‐value compared to untreated (*P < 0.05, **P < 0.01, ***P < 0.001).

To further explore whether AXL is an effective therapeutic target for preventing adaptive resistance in *FLT3*/ITD AML, we inhibited AXL with two different inhibitors, TP‐0903 or R428, concurrently with FLT3 TKI treatment [45, 46, 47]. To determine the effect of AXL inhibition on already‐reactivated ERK, cells were treated with FLT3 TKI for 24 h first to induce AXL upregulation and pERK rebound, followed by treatment with FLT3 TKI alone, R428 alone, or the combination for 1 h (Fig. 3A,B). Both R428 alone and in combination with FLT3 TKI decreased pAXL and pERK signal, with the combination resulting in increased ERK inhibition. Next, cells were treated with AXL inhibitor TP‐0903 concurrently with FLT3 TKI for 24 h (Fig. 3C,D). Combined treatment resulted in decreased pERK reactivation compared to treatment with either TKI alone. Of note, combined treatment with TP‐0903 also reduced total AXL (Fig. 3C,D). The suppression of AXL protein levels by TP‐0903 was previously observed and could be part of the drug's mechanism of action [48]. Combined AXL and FLT3 inhibition also lowered pAKT levels (Fig. 3A–D), demonstrating that combining FLT3 and AXL inhibitors can result in increased suppression of downstream pathways other than the ERK pathway.

To next explore whether AXL inhibition can further sensitize *FLT3*/ITD cells to FLT3 TKI treatment, Molm14 and MV4;11 cells were treated with a range of drugs and combinations and assayed for both proliferation (Fig. 3E,F, Fig. S4A) and apoptosis (Fig. 3G,H, Fig. S4B). The combination of R428 with FLT3 TKI significantly decreased cell proliferation and increased apoptosis compared to either drug alone (Fig. 3E,G, Fig. S4A,B). The combination index (CI) values calculated indicate that R428 and FLT3 inhibitors synergize with each other to reduce proliferation and increase apoptosis (Table S1). Additionally, the dose reduction index (DRI), which demonstrates how many fold the dose of a drug is reduced in a synergistic combination, showed how the IC50 doses of FLT3 TKI are reduced by as much as 15‐fold when combined with R428 versus gilteritinib or sorafenib alone (Fig. S4C). Treatment with TP‐0903 alone did not have a strong effect on proliferation at lower concentrations, but the combination with sorafenib strongly decreased proliferation (Fig. 3F, Fig. S4A). TP‐0903 also synergized with FLT3 TKI to increase apoptosis (Fig. 3H, Fig. S4B, Table S1). Once again, the DRI values indicated a reduction of the IC50 for FLT3 TKI in combination with TP‐0903 (Fig. S4C).

**Fig. 4:**
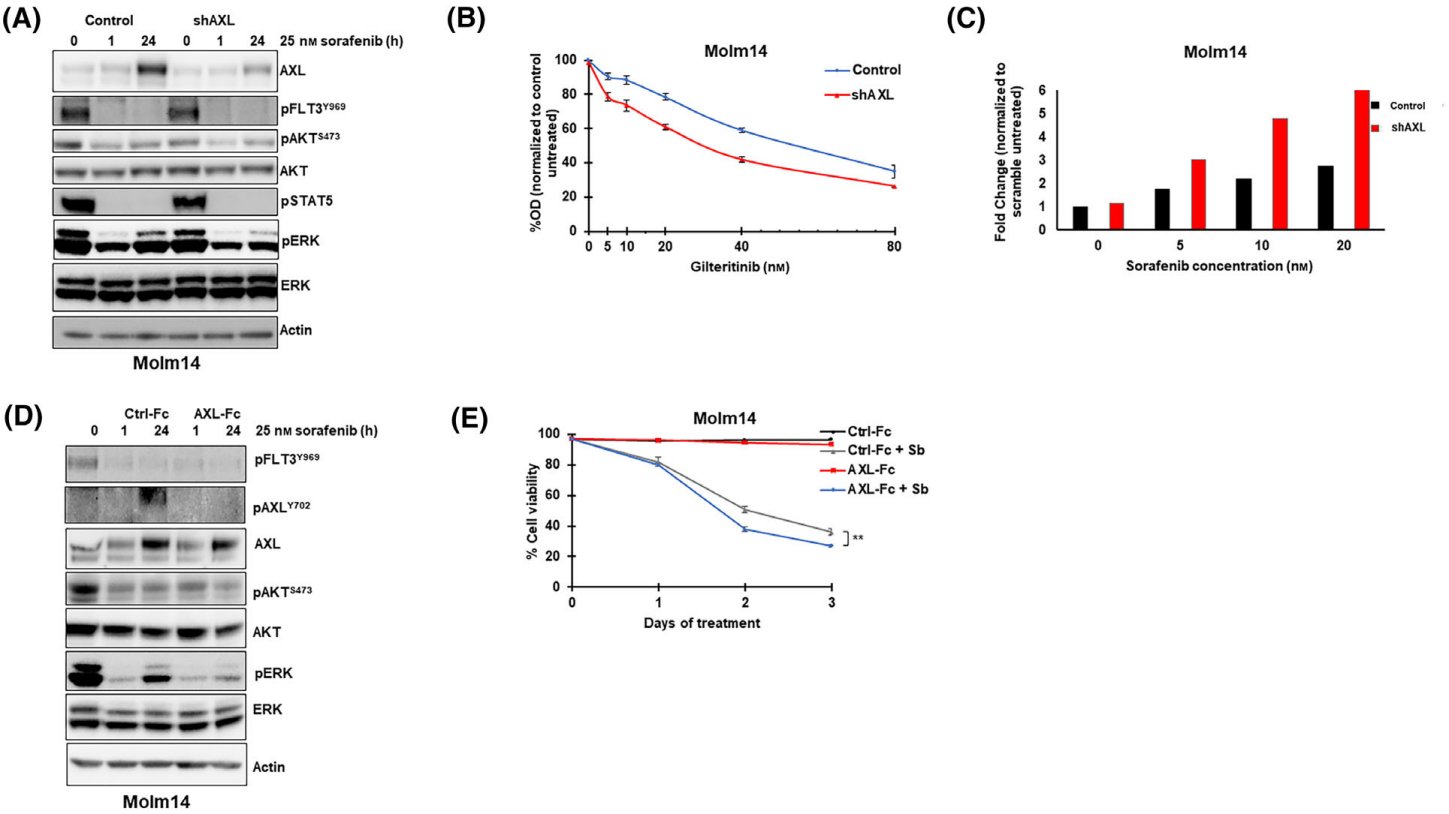
Genetic knockdown of AXL and ligand‐trapping decreases pERK reactivation and sensitizes cells to FLT3 tyrosine kinase inhibitor treatment. (A) Molm14 cells stably transfected with either a non‐silencing control vector (control) or with an shRNA construct targeting AXL (shAXL) were treated with 25 nm of sorafenib and 0.5 μg·mL^−1^ of doxycycline for 24 h. Protein lysates were subjected to immunoblot analysis against the indicated antibodies. The blots are representative of two independent experiments. (B) Molm14 cells transduced with either shAXL or control constructs were treated with 5–80 nm of gilteritinib and 0.5 μg·mL^−1^ of doxycycline concurrently for 48 h. Proliferation was analyzed with 3‐(4,5‐dimethylthiazol‐2‐yl)‐5‐(3‐carboxymethoxyphenyl)‐2‐(4‐sulfophenyl)‐2H‐tetrazolium MTS assay in triplicate. Error bars indicate average percent OD ± SD (C) shAXL and control Molm14 cells were treated with the indicated concentrations of sorafenib and 0.5 μg·mL^−1^ of doxycycline concurrently for 48 h. Apoptosis was measured with Annexin V assay (*N* = 1). (D) Molm14 cells were treated with 25 nm of sorafenib and either 4 μg·mL^−1^ of Ctrl‐Fc or AXL‐Fc for 24 h. Protein lysates were assayed by immunoblot analysis against the indicated antibodies. The blots are representative of two independent experiments. (E) Molm14 cells were treated with the indicated drug combinations (sorafenib concentration: 25 nm, Ctrl‐Fc and AXL‐Fc concentrations: 1 μg·mL^−1^) for a total of 3 days. Cells were counted at the indicated time points and percent cell viability was assessed using trypan blue exclusion assay. The experiments were done in triplicate and the error bars show the average percent viability ± SD. *P* values were determined using the unpaired student *t*‐test (**P < 0.01). Sb, sorafenib.

Pharmacological inhibition of AXL signaling combines with FLT3 TKI to inhibit pERK rebound and enhance antileukemic effects. However, TKIs also have off‐target effects on other kinases, especially as their concentration is increased. For example, TP‐0903 can inhibit Aurora kinases A and B and JAK2, and although R428 is considered a more selective AXL inhibitor, it also binds to HER2, EGFR, and other kinases [45, 49]. Therefore, to confirm in a more specific way that the observed ERK rebound is at least partially due to AXL signaling, we sought to inhibit AXL by using both shRNA knockdown and an AXL‐Fc chimeric receptor (AXL‐Fc) that acts as a ligand trap. The introduction of doxycycline‐inducible shRNA constructs against AXL (shAXL and shAXL‐2) to Molm14 cells resulted in knockdown of AXL levels in response to doxycycline treatment in the shAXL cells (Fig. S5A)Twenty‐four hour treatment with a FLT3 TKI and doxycycline decreased the extent of AXL induction in the AXL knockdown cells compared to the non‐silencing control (Fig. 4A, Fig. S5B). In response, the FLT3‐TKI treated shAXL cells showed decreased pERK rebound compared to treated control cells, although it did not completely eliminate the rebound. Knocking down AXL protein levels resulted in decreased cellular proliferation and increased apoptosis when the cells were treated with a FLT3 TKI (Fig. 4B,C).

Twenty‐four hour treatment of Molm14 and MV4;11 with AXL‐Fc and FLT3 TKI together resulted in sustained inhibition of pAXL signal compared to treatment with FLT3 TKI and a control IgG chimeric receptor (Fig. 4D, Fig. S5C). Again, decreased pERK rebound was observed in the AXL Fc‐treated cells compared to the control cells. Concurrent treatment with AXL‐Fc and FLT3 TKI for 48 h resulted in decreased cell viability compared to treatment with TKI alone (Fig. 4E, Fig. S5D).

Taken together, the data demonstrate that AXL inhibition, whether through small‐molecule inhibition, genetic knockdown, or ligand trapping, results in decreased pERK reactivation and sensitizes *FLT3*/ITD cells to FLT3 TKI treatment. This further validates AXL as a therapeutic target to prevent adaptive ERK reactivation.

### 3.4 Combined AXL and FLT3 inhibition combats adaptive resistance through impeding leukemic cell recovery

**Fig. 5:**
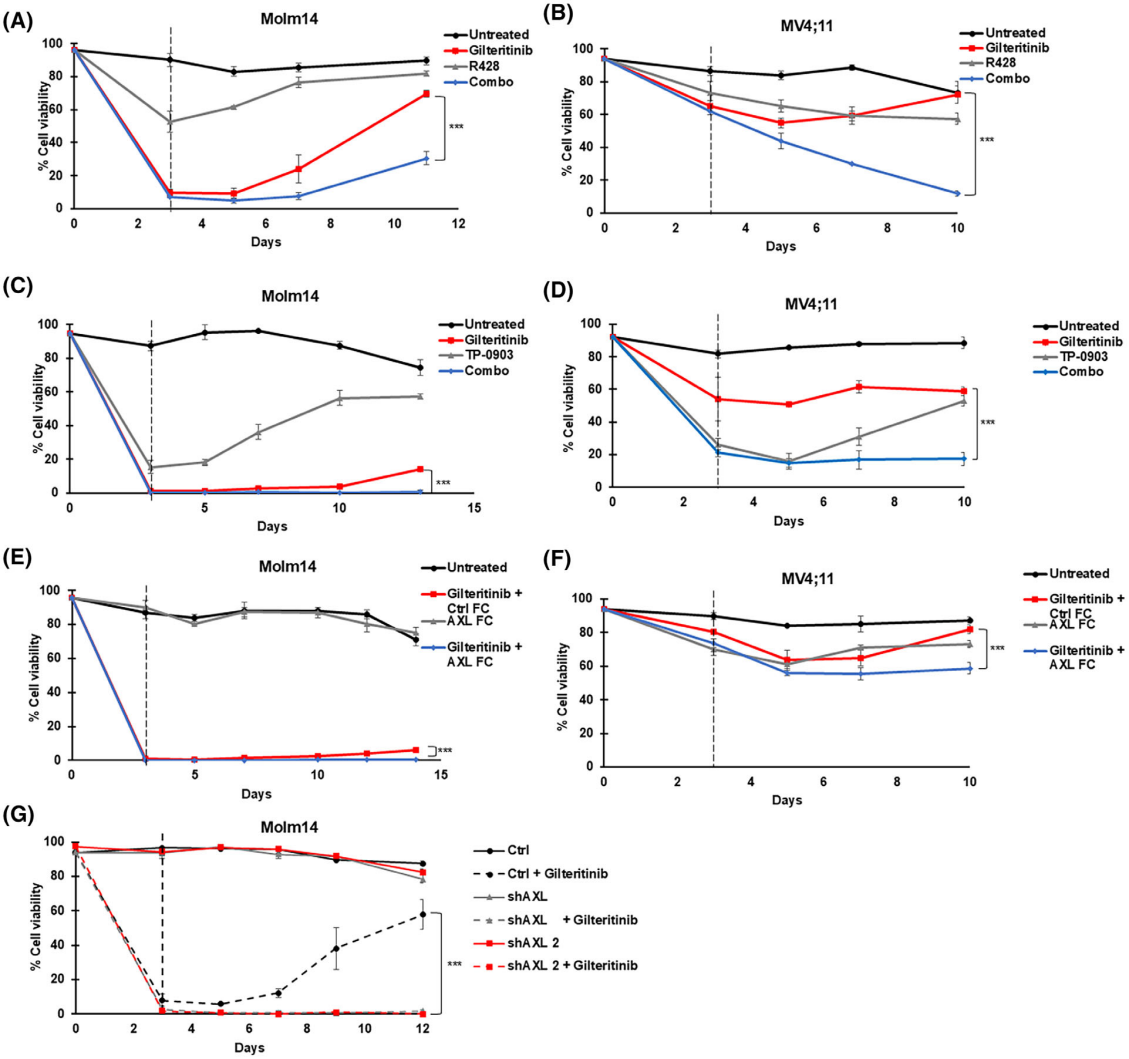
FLT3 and AXL inhibition combines to impede leukemic cell recovery after initial transient drug exposure. (A) Molm14 and (B) MV4;11 cells were treated with 75 nm of Gilteritinib, 500 nm of R428, and the combination for 72 h. (C) Molm14 and (D) MV4;11 cells were treated with 100 nm of gilteritinib, 25 nm of TP‐0903 or the combination for 72 h. (E) Molm14 and (F) MV4;11 cells were treated with 100 nm gilteritinib, or 1 μg·mL^−1^ of Ctrl‐Fc or 1 μg·mL^−1^ of AXL‐Fc, or the combination of 100 nm gilteritinib with 1 μg·mL^−1^ of AXL‐Fc or 1 μg·mL^−1^ of Ctrl‐Fc for 72 h. (G) Molm14 cells stably transfected with either an inducible non‐silencing control vector (control) or with inducible shRNA constructs targeting AXL (shAXL and shAXL‐2) were treated with 0.5 μg·mL^−1^ of doxycycline alone or 100 nm of gilteritinib for 72 h. After the 72‐h exposure, the cells were replated in fresh media and percent cell viability was evaluated at the indicated time points using Trypan Blue exclusion assay. The dotted lines indicate when the cells were replated in fresh media. The experiments were done in biological triplicate and the error bars indicate the mean of those triplicates ± SD. *P* values were determined using the unpaired student *t*‐test (***P < 0.001).

The ultimate result of adaptive response causing reactivation of downstream signals is the enabling of cancer cells to survive initial drug treatment and therefore potentially progress to other forms of resistance [17, 50]. Melgar et al. [51] characterized adaptive resistance in *FLT3*/ITD cells through the recovery of leukemic cell growth after treatment with FLT3 TKI for 72 h, and they found that after cessation of TKI treatment, AML cells recovered cell viability and resumed growth Therefore, we wanted to evaluate the effect of AXL inhibition on the persistence and recovery of *FLT3*/ITD AML cells after TKI treatment. We treated Molm14 and MV4;11 cells with FLT3 TKI alone, AXL inhibitors alone, and the combination for 72 h, then replated in fresh media and evaluated cell viability over multiple days (Fig. 5A–F). The shAXL inducible knockdown and control lines were also treated with gilteritinib and doxycycline for 72 h and then released from treatment (Fig. 5G). Treatment with a FLT3 inhibitor alone resulted in an initial decrease in cell viability after drug exposure followed by a recovery of cell viability, similar to the observations previously reported [51]. The combination of FLT3 and AXL inhibition resulted in lower cell viability after 72 h of treatment compared to FLT3 TKI alone, and the cells sustained low levels of viability after several days of plating in fresh media, with the combination severely suppressing recovery. These results indicate that the combination of FLT3 and AXL inhibition prevented leukemia cell recovery for several days after stopping treatment, highlighting how this combination can have lasting effects on leukemia cells and has the potential to improve treatment outcomes.

### 3.5 TKI‐dependent AXL upregulation is observed in human primary AML cells and other RTK‐driven cancers

**Fig. 6:**
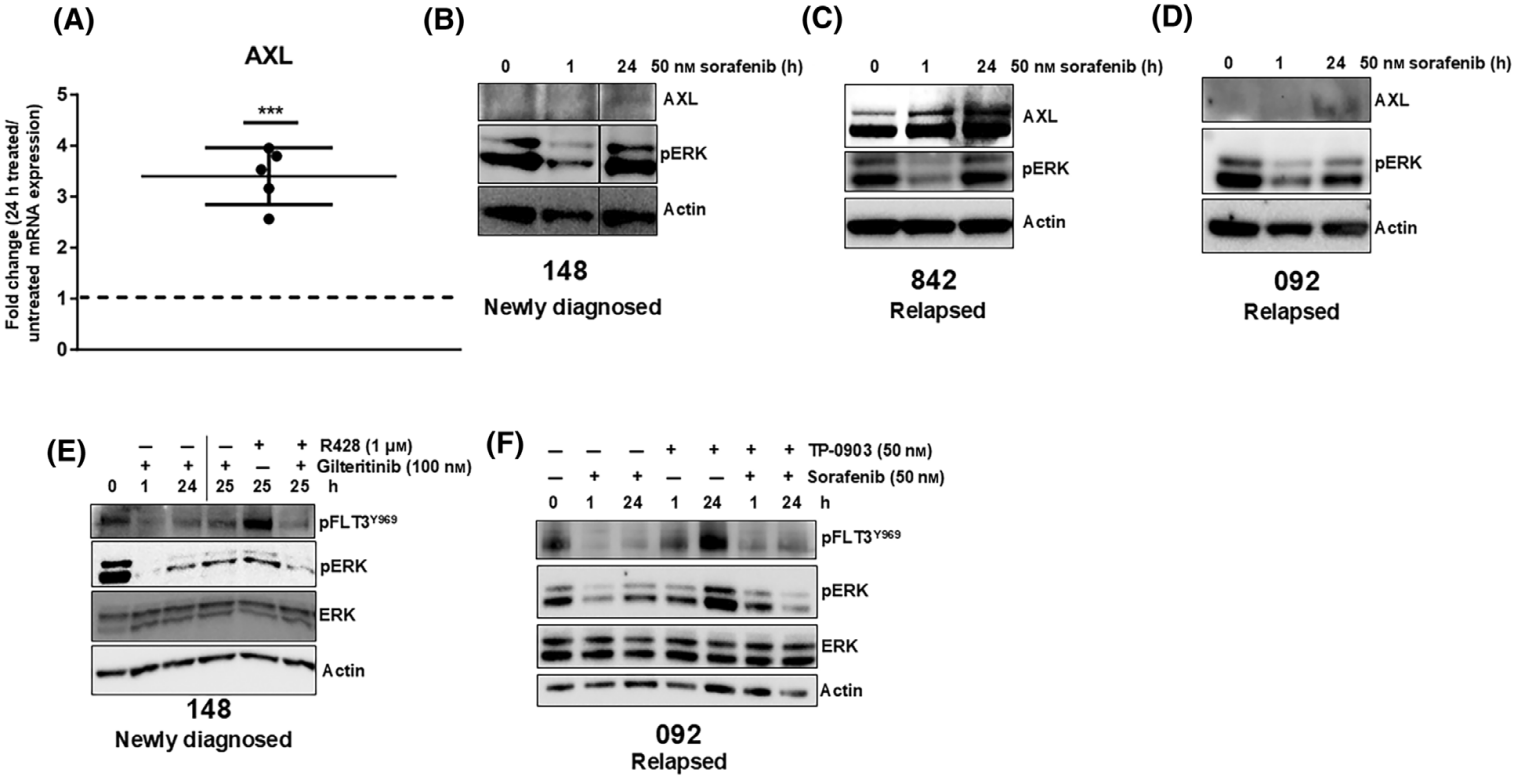
Combined AXL and FLT3 inhibition decreases ERK signaling in patient blasts. (A) Blasts from five patient samples (newly diagnosed samples 863, 310, 336, and 431; and relapsed sample 092) were treated either with or without 50 nm sorafenib for 24 h. AXL mRNA expression was evaluated in the untreated and treated samples using quantitative RT‐PCR on triplicate samples normalized to GAPDH. The fold change representing the 24‐h treated expression versus the untreated expression was plotted on the graph, with the error bar representing the average fold change of treated vs. untreated ± SD. The dotted line on the 1 indicates the normalized untreated expression for the patient blasts. The *P*‐value was evaluated using a one‐sample *t*‐test against the value of 1 (***P < 0.001). (B–D) Patient samples 148 (B), 842 (C), and 092 (D) were treated with 50 nm of sorafenib for 24 h. Protein lysate was collected at the indicated time points and analyzed with immunoblot assay. The black lines intersecting the blots in (B) indicate that the bands on either side of the line were not originally next to each other on the original immunoblot. (E) Patient sample 148 was treated with 100 nm gilteritinib for 24 h, after which the drug was washed out and replaced with the indicated drug combinations for 1 h. The black line indicates when the drug was washed out. Protein lysate was analyzed by immunoblot assay. (F) Relapsed patient sample 092 was treated with the indicated drugs for 24 h. Protein lysates were analyzed by immunoblot assay. The blots are representative of two independent experiments.

We observed AXL upregulation in *FLT3*/ITD AML cell lines in response to FLT3 TKI treatment, and targeting this upregulation appears to reduce much of the observed adaptive ERK reactivation in *FLT3*/ITD cell lines. Because immortalized cell lines sometimes differ from primary cells and thus can respond differently to drugs [52], it is important to confirm these findings in primary cells. Our previous work showed that primary *FLT3*/ITD leukemic cells also exhibit pERK rebound when treated with FLT3 TKI [14]. Thus, we examined whether AXL upregulation also occurs in primary *FLT3*/ITD AML samples. Primary *FLT3*/ITD leukemic blasts (Table S2) from patients with either newly diagnosed or relapsed disease were treated for 24 h with sorafenib. In both newly diagnosed and relapsed samples, we observed TKI‐induced AXL upregulation at both the mRNA and protein level, along with the pERK rebound observed in previous work [14] (Fig. 6A–D). Due to the differences in quantity and quality within patient samples, it was difficult to acquire as strong an AXL signal as in our cell lines. We next examined the effect of combined AXL and FLT3 inhibition on reactivated ERK signaling. The combination treatment decreased the pERK signal and cell viability compared to either drug alone in both relapsed and newly diagnosed samples, confirming AXL's role in the adaptive response observed in primary leukemic blasts (Fig. 6E,F, Fig. S6). Overall, this data further validates the role that AXL plays as a therapeutic target against adaptive resistance in *FLT3*/ITD AML patients and highlights the potential for the combination of AXL and FLT3 inhibitors to improve clinical outcomes.

Adaptive response to TKI treatment through ERK reactivation has been observed by us and others in malignancies other than AML [14, 50, 53]. Thus, we wanted to see if TKI‐mediated AXL upregulation and activation also occurs in other RTK‐driven cancers that exhibit pERK rebound. We treated the HER2‐amplified SKBR3 breast cancer line with lapatinib and the *EGFR*‐mutated HCC827 lung cancer line with erlotinib and combined those treatments with AXL inhibitors. We found that SKBR3 breast cancer cells experienced TKI‐induced AXL upregulation, as measured by both protein and mRNA levels, and decreased pERK rebound in response to AXL inhibition (Fig. S7A,B). In contrast, HCC827 cells demonstrated decreased AXL protein and mRNA in response to erlotinib treatment and were not sensitive to combined EGFR and AXL inhibition (Fig. S7C,D). These results demonstrate that changes in AXL expression and signaling can occur early in the TKI treatment of multiple cancers, but AXL is unlikely to be universally responsible for adaptive resistance in all RTK‐driven cancers.

### 3.6 AXL inhibition *in vivo* combines with gilteritinib to reduce leukemia burden and improve survival

**Fig. 7:**
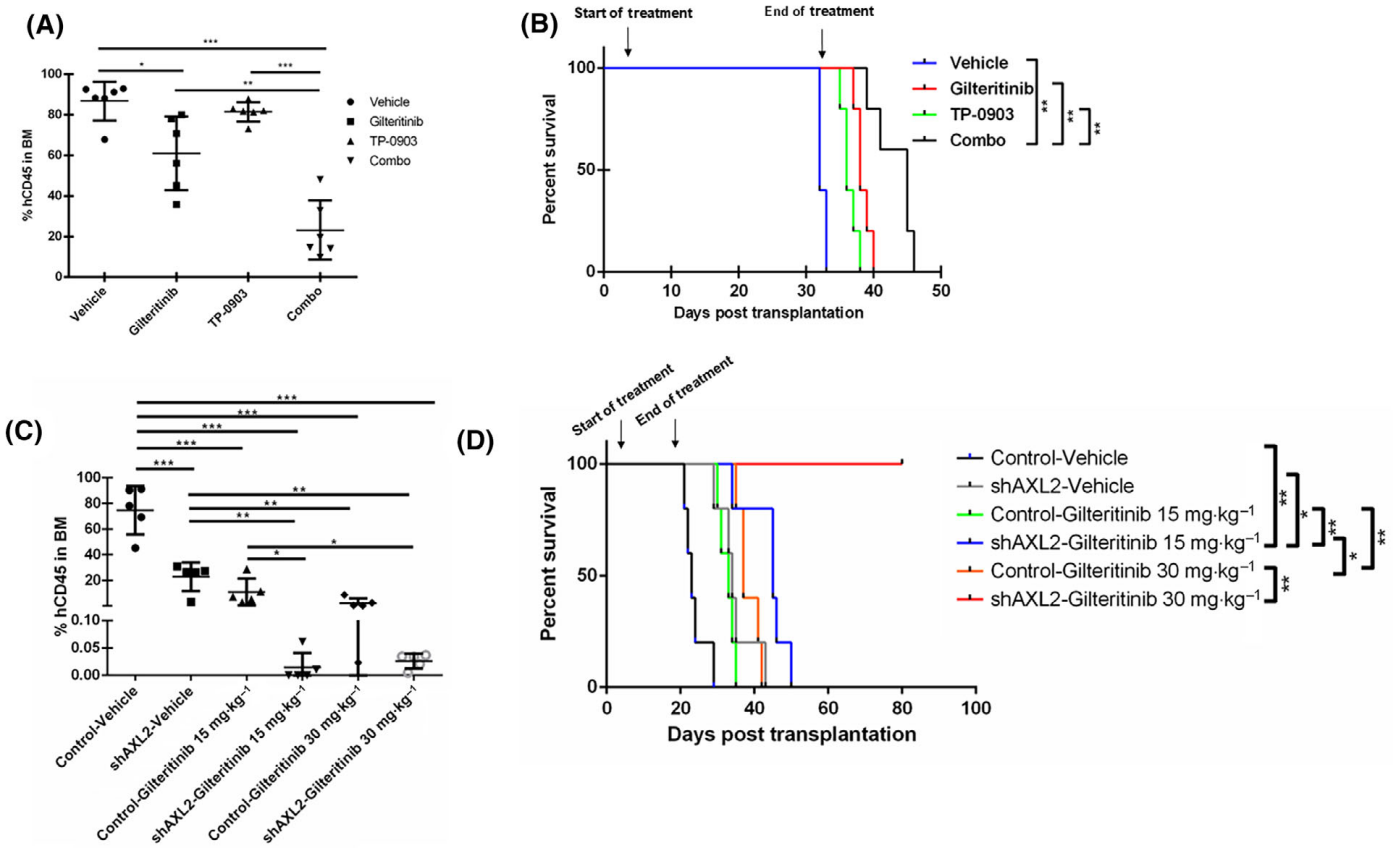
Combined AXL and FLT3 inhibition reduces leukemia burden and increases survival *in vivo*. (A) Molm14 cells were injected into NSG mice via tail vein. Seventy‐two hours after transplantation, the mice were randomized into groups and treated with either 15 mg·kg^−1^ of gilteritinib, 30 mg·kg^−1^ of TP‐0903, or the combination, once daily excluding weekends. After 4 weeks of treatment, bone marrow was collected via aspiration and evaluated for human CD45 (hCD45) positive leukemia cells. *N* for each group is ≥ 6. Error bars indicate mean ± SD, and significance was determined by unpaired student *t*‐test (*P < 0.05, **P < 0.01, ***P < 0.001). (B) Kaplan–Meier survival curves for the treatment and vehicle groups. The survival is measured in days post transplantation. The arrows above the curves indicate the start of treatment (day 3) and the end of treatment (day 31). *N* for each group is ≥ 5. Error bars indicate mean ± SD and significance was determined by long rank analysis (**P < 0.01). (C) Inducible shAXL‐2 and control Molm14 cells were injected into NSG mice. Seventy‐two hours after injection, the mice were randomized into groups and treated with either 15 mg·kg^−1^, 30 mg·kg^−1^ of gilteritinib, or vehicle once daily excluding weekends. 500 μg·mL^−1^ of doxycycline was provided daily via acidified drinking water available in the animal facility. After 23 days post injection, treatment was stopped, and bone marrow was collected via aspiration and evaluated for human CD45 (hCD45) positive leukemia cells. *N* for each group is 5. Error bars indicate mean ± SD and significance was determined by unpaired student *t*‐test (*P < 0.05, **P < 0.01, ***P < 0.001). (D) Kaplan–Meier survival curves for the shAXL and control treatment and vehicle groups. The survival is measured in days post transplantation. The arrows above the curves indicate the start of treatment (day 3) and the end of treatment (day 23). *N* for each group is = 5. Error bars indicate mean ± SD, and significance was determined by long‐rank test (*P < 0.05, **P < 0.01).

We next explored the efficacy of combined FLT3 TKI and AXL inhibition in a murine model. NOD/SCID gamma (NSG) mice were engrafted with Molm14 cells and treated with either gilteritinib, TP‐0903, or the combination for 4 weeks. The doses used for both gilteritinib and TP‐0903 were below the maximally tolerated dose of each [54, 55]. Gilteritinib treatment lowered the level of human leukemia cells compared to the vehicle‐treated mice, whereas treatment with TP‐0903 did not produce significant changes (Fig. 7A). Treatment with the combination further reduced leukemia burden compared to either treatment alone. Additionally, treatment with either drug alone increased survival, and the mice treated with the combination of both drugs survived the longest (Fig. 7B).

Next, NSG mice were implanted with either inducible shAXL knockdown cells or non‐specific control cells and treated with either 15 or 30 mg·kg^−1^ of gilteritinib and with doxycycline for 4 weeks. The results mirror the drug combination experiment, with the shAXL mice treated with gilteritinib having lower leukemia burden, and increased survival compared to the control mice treated with gilteritinib alone or vehicle alone (Fig. 7C,D). The shAXL mice treated with 15 mg·kg^−1^ gilteritinib survived significantly longer than even the control mice treated with 30 mg·kg^−1^ gilteritinib. The shAXL mice treated with 30 mg·kg^−1^ of gilteritinib survived for weeks longer than any other group (Fig. 7D), and at the time of this report, the entire cohort were still alive and looking well 57 days after terminating therapy. In addition, 21 days after stopping treatment, we reevaluated leukemia burden in the bone marrow of the surviving mice and found that the shAXL mice treated with 30 mg·kg^−1^ of gilteritinib still had very low leukemia burden (less than 3%), highlighting that combined AXL and FLT3 inhibition has striking effects on *in vivo* survival (Fig. S8A). Even the shAXL knockdown with vehicle group had comparable leukemia burden and survival to the control group treated with 15 mg·kg^−1^ of gilteritinib, suggesting that AXL knockdown alone reduces leukemic progression (Fig. 7C,D). This is supported by other work [56, 57]. Finally, to evaluate the potential effects of the AXL/FLT3 combined inhibition on normal hematopoietic toxicity, we treated healthy human CD34+ PBSCs with AXL and FLT3 inhibitors and subjected them to colony formation assays (Fig. S8B). Both the combinations and each drug alone showed no difference in colony forming ability compared to the untreated control, indicating that this combination spares hematopoiesis in healthy cells, an important consideration when evaluating an antileukemic therapy. Collectively, these data provide further *in vivo* evidence that combining AXL and FLT3 inhibition has the potential to improve treatment efficacy in *FLT3*/ITD AML.

## 4 Discussion

FLT3 inhibition using FLT3 TKIs continues to be a promising approach against *FLT3*/ITD AML. However, the short‐lived clinical responses of these inhibitors point to multiple mechanisms of resistance [58, 59]. Previous work in our lab showed that FLT3 inhibition induces an adaptive response that is discernible within 16–24 h of treatment of leukemia cells, resulting in reactivation of ERK signaling just hours after starting treatment [14]. Signaling adaptation in response to TKIs and other targeted therapies has been observed in other cancers [17, 60], indicating the need to improve our understanding of short‐term adaptive resistance and to elucidate the mechanisms underlying it. Thus, we sought to discover the pathways responsible for the ERK reactivation seen in FLT3 inhibition of *FLT3*/ITD leukemia.

The work reported here demonstrates that the RTK AXL is upregulated within 4 h after starting FLT3 TKI treatment and plays a role in pERK reactivation. AXL activation has been previously implicated in longer‐term resistance of AML and other cancers. Ben‐Batalla et al. showed that patients with higher levels of AXL mRNA had worse prognosis, and IK Park et al. found that AXL is upregulated in TKI‐resistant cell lines [32, 33]. In addition, ectopic AXL overexpression and activation in AML cells resulted in increased ERK and AKT signaling and increased resistance to chemotherapy agents [61]. Other papers have also linked AXL upregulation to acquired resistance [49, 62]. This report links induced AXL expression as an almost immediate adaptive response to FLT3 TKI treatment and links it to ERK signaling reactivation.

It was interesting that treatment with gilteritinib, touted as a dual AXL/FLT3 inhibitor, still induced AXL upregulation, even at higher concentrations. Gilteritinib does decrease AXL signaling in a dose‐dependent manner. However, AXL upregulation and activation still occurs at concentrations that effectively inhibit FLT3, and combined treatment of gilteritinib with the AXL inhibitors R428 or TP‐0903 abolished the pERK rebound and further suppressed proliferation and increased apoptosis. Furthermore, the combination of gilteritinib and TP‐0903 had the strongest *in vivo* response compared to either drug alone. These observations suggest that AXL upregulation plays a role in ERK reactivation even in gilteritinib‐treated cells and that gilteritinib treatment alone is not enough to overcome the TKI‐induced AXL activation. This is likely because gilteritinib is 20‐fold more potent against FLT3 compared to AXL so it is unlikely that AXL would be significantly inhibited at the trough drug levels achieved in patients [39, 40]. This is very important to consider in future studies, especially when counting on gilteritinib to act as an efficient AXL inhibitor.

We also showed that combined treatment of a MEK inhibitor with a FLT3 inhibitor can abolish the pERK rebound without affecting AXL induction. Thus, the reactivation of ERK in these cell lines is not the cause of AXL upregulation at 24 h of treatment. This observation is notable since past studies reported that AXL activation and expression were lowered with MEK inhibition in AML cell lines [33, 63]. Furthermore, AXL upregulation in cancer resistance has been linked to the AP‐1 transcription family, which can be activated downstream of ERK signaling [28, 64]. It is possible that the ERK pathway and AP‐1 transcription family can still play a role in FLT3 TKI‐induced AXL activation, but the AXL upregulation that occurs within 24 h after TKI treatment does not appear to be the result of ERK signaling.

On the other hand, we demonstrated that inhibiting the YAP/TAZ pathway along with FLT3 lowered AXL expression, suggesting that YAP may play a role in FLT3 TKI‐induced AXL upregulation. The YAP/TAZ pathway is linked to AXL upregulation in cancer, and YAP has even been implicated as a driver of adaptive resistance to KRAS inhibitors [65]. Therefore, it is possible that targeting this pathway may sensitize FLT3 TKI treatment against adaptive resistance, providing an exciting future avenue of investigation. AXL upregulation in cancer has been linked to transcription factors SP‐1 and MZF1, as well as STAT5 activation and hypoxia [28, 64, 66]. In this report, we observed that STAT5 phosphorylation was decreased throughout FLT3 TKI treatment, but more work needs to be done to further determine the mechanisms behind the early AXL upregulation after FLT3 inhibition.

We determined that the combination of FLT3 TKI with AXL inhibition resulted in decreased ERK reactivation and sensitized *FLT3*/ITD AML cells to FLT3 TKI treatment. *In vivo* experiments demonstrated further reduction of leukemic cells and increased survival in mice treated with the drug combination and injected with the AXL knockdown cells. Higher doses of each drug might prove even more efficacious [54, 55], especially since the normal CD34+ studies indicated that the AXL and FLT3 combination spares hematopoiesis in healthy cells. In other literature, AXL inhibition with both drugs and genetic silencing had little effect on normal hematopoiesis in both human and murine CD34+ cells [49, 55, 67]. A comparison between gilteritinib and quizartinib revealed that gilteritinib treatment had a lower toxicity towards normal murine hematopoiesis in an AML‐impaired bone marrow microenvironment [68]. This and our own data suggest that the combination of FLT3 and AXL inhibitors may have a large therapeutic window due to reduced hematopoietic toxicity. Currently, a variety of AXL‐targeting agents, including small molecules, monoclonal antibodies, antibody‐drug conjugates, and soluble receptors, are in various stages of clinical trials in both solid tumors and AML. They are being investigated as monotherapies or in combination with other therapeutics including chemotherapies, targeted therapies, and even immune checkpoint inhibitors. R428 is perhaps the best studied inhibitor, and its role in NSCLC is very encouraging with clinical response achieved when paired with chemotherapy, EGFR TKI, or immune checkpoint inhibitors [69]. Regarding clinical trials in AML, R428 is currently being investigated in phase IB/II clinical trials in patients with relapsed/refractory AML (NCT02488408). TP‐0903 underwent and completed its own phase IB/II clinical trial in FLT3‐mutated patients (NCT04518345). Neither clinical trial includes testing the AXL inhibitor with FLT3 TKI in *FLT3*/ITD AML patients, despite ours and other preclinical data demonstrating that combining FLT3 and AXL inhibitors sensitizes *FLT3*/ITD AML cells to FLT3 TKI treatment [33, 66, 70]. We believe the future of targeting AXL in AML involves a combinatorial approach with other targeted therapies, and our data further validate this idea and demonstrates the importance of combining AXL inhibition concurrently with FLT3 TKI to combat early adaptive resistance as well as later‐occurring acquired resistance.

Finally, it is likely that pathways other than AXL signaling also play roles in the adaptive resistance observed in *FLT3*/ITD AML. Rapid ERK adaptation observed in many cancers has been attributed to ERK‐dependent feedback inhibition, which induces RTK expression and activation and reprograms the signaling network [17, 60]. As such, cancers that experience adaptive resistance against targeted therapies often have multiple RTKs contributing to the adaptation [71]. Recently, Melgar et al. [51] demonstrated that the activation of innate immune pathways contributes to adaptive resistance in *FLT3*/ITD AML, indicating that pathways outside of RTK signaling can contribute to adaptive resistance. These findings suggest that AXL signaling is likely not the sole driver of ERK reactivation and that adaptive resistance should continue to be investigated to find additional strategies to improve treatment for patients with *FLT3*/ITD AML.

## 5 Conclusions

This study expands on our previous work demonstrating an adaptive ERK reactivation response in *FLT3*/ITD AML. Adaptive resistance often relies on the activation of alternative RTKs to reactivate downstream pathways, and we hypothesized that targeting AXL may overcome the adaptive response. The following experiments reveal that FLT3 TKI treatment causes rapid AXL upregulation in *FLT3*/ITD cell lines and in patient samples. This upregulation is associated with ERK reactivation, and concurrent FLT3 and AXL inhibition diminishes adaptive ERK reactivation and sensitizes leukemia cells to FLT3 TKI treatment. The combination also impeded leukemic cell recovery and reduced leukemia burden *in vivo*. While past studies have linked AXL to long‐term acquired resistance in *FLT3*/ITD AML, our data show that AXL upregulation can have an impact mere hours after starting FLT3 TKI treatment. Taken altogether, our data suggest that AXL plays a role in adaptive resistance in *FLT3*/ITD AML and that targeting AXL along with FLT3 can improve patient outcomes.

## Conflict of interest

DS serves on the SAB of InSilico Medicine and consults for Pharos I&BT Co., Ltd. MJL has received honoraria from Daiichi Sankyo, Novartis, and Agios; has served in a consulting or advisory role for Daiichi Sankyo, Novartis, and Agios; and has received research funding from Astellas and Novartis. CAP receives research support from Kura Oncology and Novartis Institute for Biomedical research and has served in a consulting role for Genentech and Day One Therapeutics. The rest of the authors declare no competing financial interests.

## Author contributions

TSS designed and performed the experiments, analyzed data and wrote the manuscript. LL designed and performed experiments, analyzed data, and revised the manuscript. JKB and MC designed and performed experiments. JS, BN, and RZ analyzed and interpreted data. MJL provided patient samples. CAP designed experiments and revised the manuscript. DS directed the project, designed experiments, analyzed data, and revised the manuscript.

### Peer review

The peer review history for this article is available at https://www.webofscience.com/api/gateway/wos/peer‐review/10.1002/1878‐0261.13749.

## Supporting information


**Fig. S1.** FLT3 TKI treatment increases AXL activation and lowers GAS6 levels in *FLT3*/ITD cell lines.
**Fig. S2.** AXL overexpression increases ERK phosphorylation and proliferation.
**Fig. S3.** AXL levels are unaffected by MEK inhibition but are reduced by PI3K and YAP inhibition.
**Fig. S4.** Combination FLT3 TKI and AXL inhibitors decrease proliferation and increase apoptosis.
**Fig. S5.** AXL inhibition through genetic knockdown and ligand trapping affects pERK rebound.
**Fig. S6.** AXL and FLT3 inhibition decrease *FLT3*/ITD primary cell viability.
**Fig. S7.** TKI‐induced AXL perturbations are observed in other RTK‐driven cancers.
**Fig. S8.** Recovery leukemia burden of shAXL knockdown mice.
**Table S1.** Combination Index (CI) values for FLT3 TKI in combination with AXL inhibitors.
**Table S2.** AML patient characteristics.

## Data Availability

Relevant data supporting this study are available within the article and its Supporting Information or from the corresponding author upon reasonable request.
