# Supplementary material for: Targeting rapid TKI‐induced AXL upregulation overcomes adaptive ERK reactivation and exerts antileukemic effects in FLT3/ITD acute myeloid leukemia
Source: Mol Oncol. 2024 Oct 12;19(5):1386–403. doi: 10.1002/1878-0261.13749 (PMC12077278; doi:10.1002/1878-0261.13749)

Supplementary Materials for

Targeting rapid TKI-induced AXL upregulation overcomes adaptive ERK reactivation and exerts antileukemic effects in *FLT3*/ITD acute myeloid leukemia

Tessa S. Seale, Li Li, J. Kyle Bruner, Melody Chou, Bao Nguyen, Jaesung Seo, Ruiqi Zhu, Mark J. Levis, Christine A. Pratilas, and Donald Small

Correspondence to: donsmall@jhmi.edu

This PDF file includes:

Supplementary Figure Legends

Figures. S1 to S8

Tables S1 to S2

Supplementary Figure Legends

Supplementary Table 1. **Combination Index (CI) values for FLT3 TKI in combination with AXL inhibitors**. Data was collected 48 hours after treatment, with the exception of MV4;11 gilteritinib and TP-0903 Annexin V combination, which was 72 hours after treatment. CI values were calculated at ED50 using CompuSyn software.

Supplementary Table 2. **AML Patient Characteristics**

Supplementary Figure 1. **FLT3 TKI treatment increases AXL activation and lowers GAS6 levels in *FLT3*/ITD cell lines.** MV4;11 cells were treated with 25 nM of sorafenib for 48 hours. Protein lysates were collected at 0 hour and 48 hours and assayed with PathScan® RTK Antibody Array panels (A). (B) The signal intensities of untreated and 48-hour duplicates were quantified and adjusted against the local background using Image Lab software. Next, the adjusted signal intensity was first normalized against the positive control intensities on each array, and then the 48 hour was normalized against the untreated. Error bars indicate average

signal intensity ± SD. (C) Molm14 cells were treated with 25 nM of sorafenib for 24 hours. Protein lysates were subjected to immunoblot analysis against the indicated antibodies. (D) MV4;11 and Molm14 cells were treated with 25 nM of sorafenib for 24 hours and GAS6 mRNA expression was measured at the indicate time points using quantitative RT-PCR as normalized to GAPDH. Error bars indicate average mRNA expression ± SEM. (E) The indicated cell lines are treated with 100 nM of gilteritinib or 10 nM of quizaritinib for 24 hours. Protein lysates are collected at the indicated time points and subjected to immunoblot analysis. CK means cytokines, and + Ctrl means positive control, which is Molm14 cells are treated with 100 nM of gilteritinib for 24 hours.

Supplementary Figure 2. **AXL overexpression increases ERK phosphorylation and proliferation** (A) Molm14 cells transduced with either a control vector (Control) or a constitutive AXL overexpression vector (AXL+) were treated with the indicated drugs for 4 hours. Protein lysates were collected at the indicated time points and subjected to immunoblot analysis. The black lines intersecting the blots indicate that the bands on either side of the line were not originally next to each other on the original immunoblot. (B) Molm14 cells transduced with either a doxycycline inducible non-specific control (indControl) or an inducible AXL overexpression construct (indAXL+) were treated with sorafenib and 0.5 µg/mL of doxycycline for 8 hours. The protein lysates were collected at the indicated time points and subjected to immunoblot analysis. The black lines intersecting the blots indicate that the bands on either side of the line were not originally next to each other on the original immunoblot. (C)) Molm14 and MV4;11 cells transduced with either a control vector (Ctrl) or a constitutive AXL overexpression vector (AXL+) were treated with the indicated concentrations of gilteritinib for 48 hours and subjected to MTT analysis. OD stands for optical density and error bars are average OD ± SD.

Supplementary Figure 3. **AXL levels are unaffected by MEK inhibition but are reduced by PI3K and YAP inhibition**. (A) Molm14 and MV4;11 cells were treated with 25 nM sorafenib and

either 10 µM of U0126 or DMSO for 24 hours. Protein lysates were collected at the indicated time points and subjected to immunoblot analysis against the indicated antibodies. The black lines intersecting the blots indicate that the bands on either side of the line were not originally next to each other on the original immunoblot. (B) Molm14 and MV4;11 cells were treated with 100 nM of gilteritinib, 3 nM of trametinib, or the combination for 24 hours and AXL mRNA expression was measured using qRT-PCR normalized to untreated control. Error bars indicated average expression ± SEM. (C) Molm14 and MV4;11 cells were treated with the indicated drug concentrations for 24 hours. Protein lysates were collected at the indicated time points and subjected to immunoblot analysis against the indicated antibodies. The line bisecting the blot indicates that the lanes were not next to each other originally and were cropped as a result. (D) MV4;11 cells transfected with either a control siRNA (ctrl) or siRNA against YAP (siYAP) were treated with 100 nM of gilteritinib for 24 hours. Protein lysates were collected at the indicated time points and subjected to immunoblot analysis against the indicated antibodies. (E, F) Molm14 and MV4;11 cells were treated with gilteritinib alone or with verteporfin (E) or VT107 (F) for 24 hours. Protein lysates were collected at the indicated time points and subjected to immunoblot analysis against the indicated antibodies. The line bisecting the blot indicates that the lanes were not next to each other originally and were cropped as a result.

Supplementary Figure 4**. Combination FLT3 TKI and AXL inhibitors decrease proliferation and increase apoptosis.** (A) MV411 and Molm14 cells were treated with the indicated drug treatments for 48 hours. Proliferation was measured by MTT assay. Error bars indicate mean percent OD ± SD. (B) MV4;11 cells were treated the indicated drugs for 72 hours. Apoptosis was assayed with Annexin V staining. Error bars indicate average fold change of % Annexin V vs. untreated ± SD. Asterisks directly above the error bar indicate P-value compared to untreated (*P <0.05, **P < 0.01, ***P <0.001). (C) Molm14 (left) and MV4;11 (right) cells were assayed with the indicated MTT or Annexin V assays with the indicated drug combinations. The

dose reduction index (DRI) was calculated for sorafenib in combination versus sorafenib alone. The dotted lines indicate the normalized sorafenib alone value.

Supplementary Figure 5. **AXL inhibition through genetic knockdown and ligand trapping affects pERK rebound.** (A) Molm14 cells stably transfected with a shRNA construct against AXL (shAXL-2) were treated either with or without 0.5 µg/mL of doxycycline for 24 hours. Protein lysates were subjected to immunoblot analysis. (B) Molm14 cells stably transfected with either a non-silencing control vector (control) or with an shRNA construct targeting AXL (shAXL-2) were treated with 25 nM of sorafenib and 0.5 µg/mL of doxycycline for 24 hours. Protein lysates were subjected to immunoblot analysis against the indicated antibodies. The blots are representative of at least two independent experiments. (C) Molm14 (left) and MV4;11 (right) cells are treated with 25 nM of sorafenib and either 3 µg/mL of Ctrl-Fc or AXL-Fc for 24 hours. Protein lysates were collected at the indicated time points and subjected to immunoblot assay. (D) MV4;11 cells were treated with the indicated drug combinations (sorafenib concentration: 25 nM, Ctrl-Fc and AXL-Fc concentrations: 1 µg/mL) for a total of three days. Cells were counted at the indicated time points and percent cell viability was assessed using trypan blue exclusion assay. The experiments were done in triplicate and the error bars show the average percent viability ± SD (**P < 0.01).

Supplementary Figure 6. **AXL and FLT3 inhibition decrease *FLT3*/ITD primary cell viability**. The indicated primary cell samples are treated with 80 nM of gilteritinib, 80 nM of TP-0903, 1 M of R428, or the combination for 24 hours. Cell viability was measured using Trypan blue exclusion assay.

Supplementary Figure 7. **TKI-induced AXL perturbations are observed in other RTK-driven cancers** (A) HER2-amplified SKBR3 cells were treated with 75 nM of lapatinib, 100 nM of TP-0903 and the combination for 24 hours. Protein lysates were analyzed with immunoblot analysis. (B) SKBR3 cells were treated with 75 nM of lapatinib. mRNA was subjected to

quantitative RT-PCR for AXL and GAS6. Expression values were measured in triplicate relative to GAPDH levels. Error bars indicate average expression ± SEM (C) EGFR-mutated HCC827 cells were treated with 100 nM of erlotinib for 24 hours, and then the cells were washed and replated and treated with either 100 nM of erlotinib, 1000 nM of R428, and the combination for an additional 1 hour. The vertical line indicates point of initial drug removal. Protein lysates were subjected to immunoblot analysis. (D) HCC827 cells are treated with 750 nM of lapatinib. mRNA was subjected to quantitative RT-PCR for AXL and GAS6 relative to GAPDH. Expression values were measured in triplicate. Error bars indicate average fold change ± SEM.

Supplementary Fig 8**. Recovery leukemia burden of shAXL knockdown mice**. Inducible shAXL-2 and control Molm14 cells were injected into NSG mice. 72 hours after injection, the mice were randomized into groups and treated with either 15 mg/kg, 30 mg/kg of gilteritinib, or vehicle once daily (excluding weekends). 500 µg/mL of doxycycline was provided daily via acidified drinking water from the animal facility. Recipients were treated for 20 days. 21 days post the end of treatment, bone marrow was collected via aspiration from surviving mice of the indicated groups and evaluated for percent of human CD45 positive leukemia cells. Error bars indicate the average % hCD45 ± SD. (B) Human CD34+ PBSCs from healthy donors are treated with gilteritinib, R428, TP-0903 or the combination and plated in methylcellulose for a colony formation assay with three biological replicates. Error bars represent average number of colonies ± SD.

Supplementary Table 1

Drug Combination	Molm14	MV4;11
**Sorafenib + R428: MTT**	0.49	0.077
**Gilteritinib + R428: MTT**	0.85	0.29
**Gilteritinib + R428: Annexin V**	0.66	0.23
**Gilteritinib + TP-0903: Annexin V**	0.61	0.34

Supplementary Table 2

Sample	Age/Gender	FLT3 mutation Status	WBC (cells/μL)	Cytogenetics	Newly-diagnosed/Relapse
863	82/F	FLT3/ITD, 96 bp insertion, VAF 46%	116,000	Normal karyotype	Newly-diagnosed
092	59/F	FLT3/ITD, 54 bp insertion, VAF 73.7%	17,600	del16q	Relapsed
148	76/F	FLT3/ITD, 60 bp insertion, VAF, 29.8%	135,600	Normal karyotype	Newly-diagnosed
842	70/M	FLT3/ITD 352 bp insertion, VAF 181%	93.750	t(4;12)(q12;p13),del(7)(q31q35)	Relapsed
310	62/F	FLT3/ITD, 364 bp insertion, VAF 55%; 349 bp insertion, VAF <5%	318,160	+8	Newly-diagnosed
431	44/F	FLT3/ITD	N/A	Normal karyotype	Newly-diagnosed
336	28/F	FLT3/ITD	N/A	Normal karyotype	Newly-diagnosed

Supplementary Figure 1

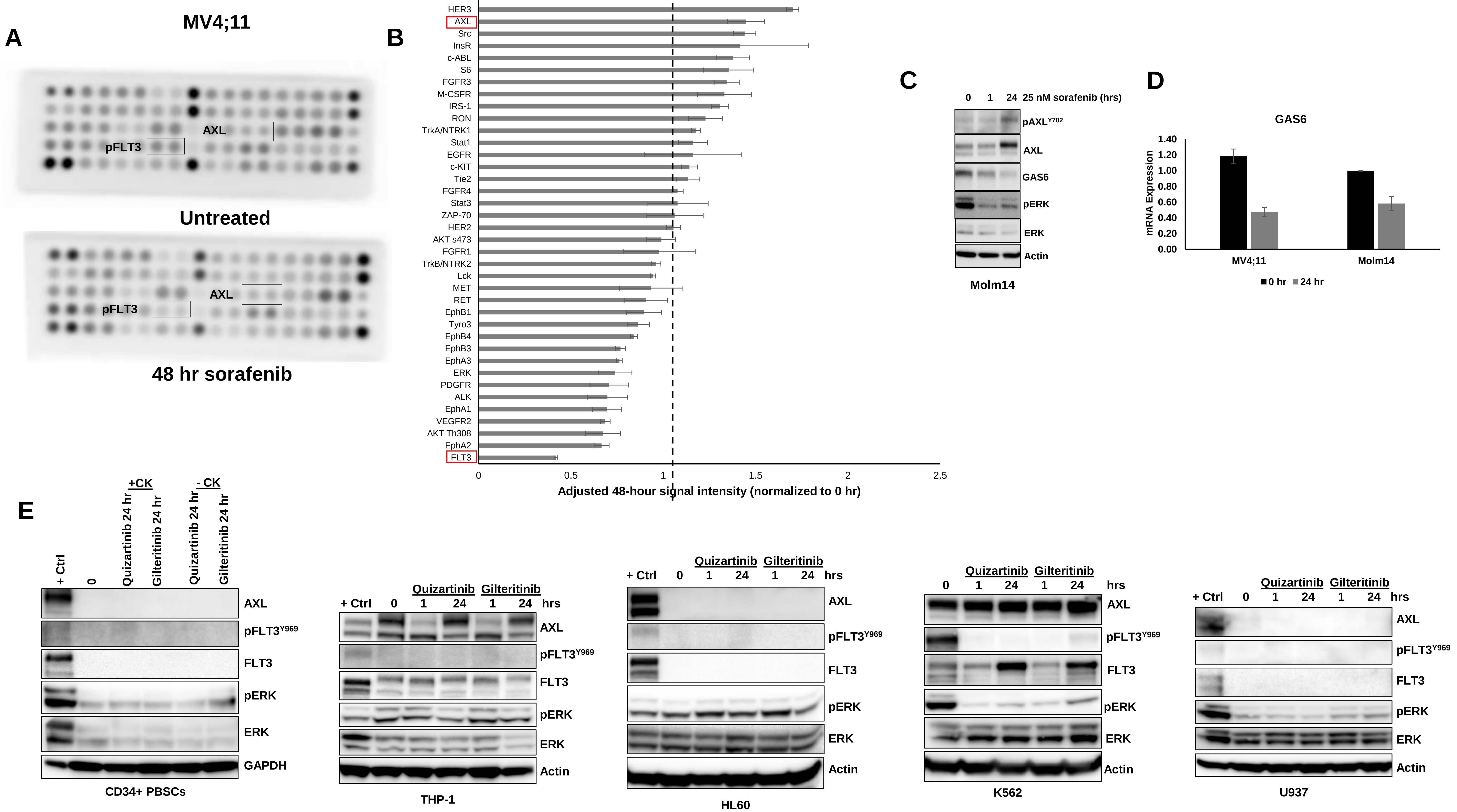

Supplementary Figure 2

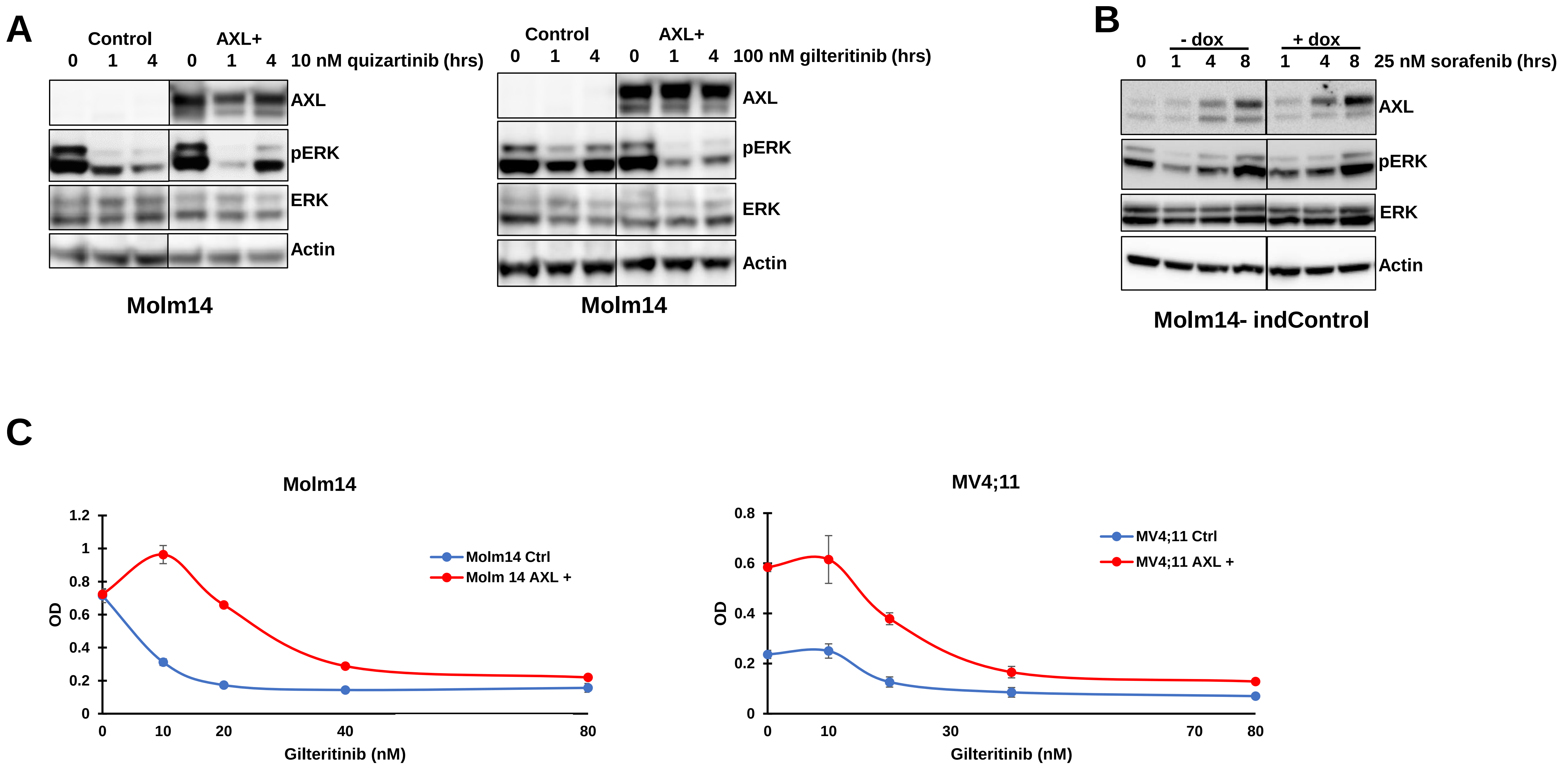

Supplementary Figure 3

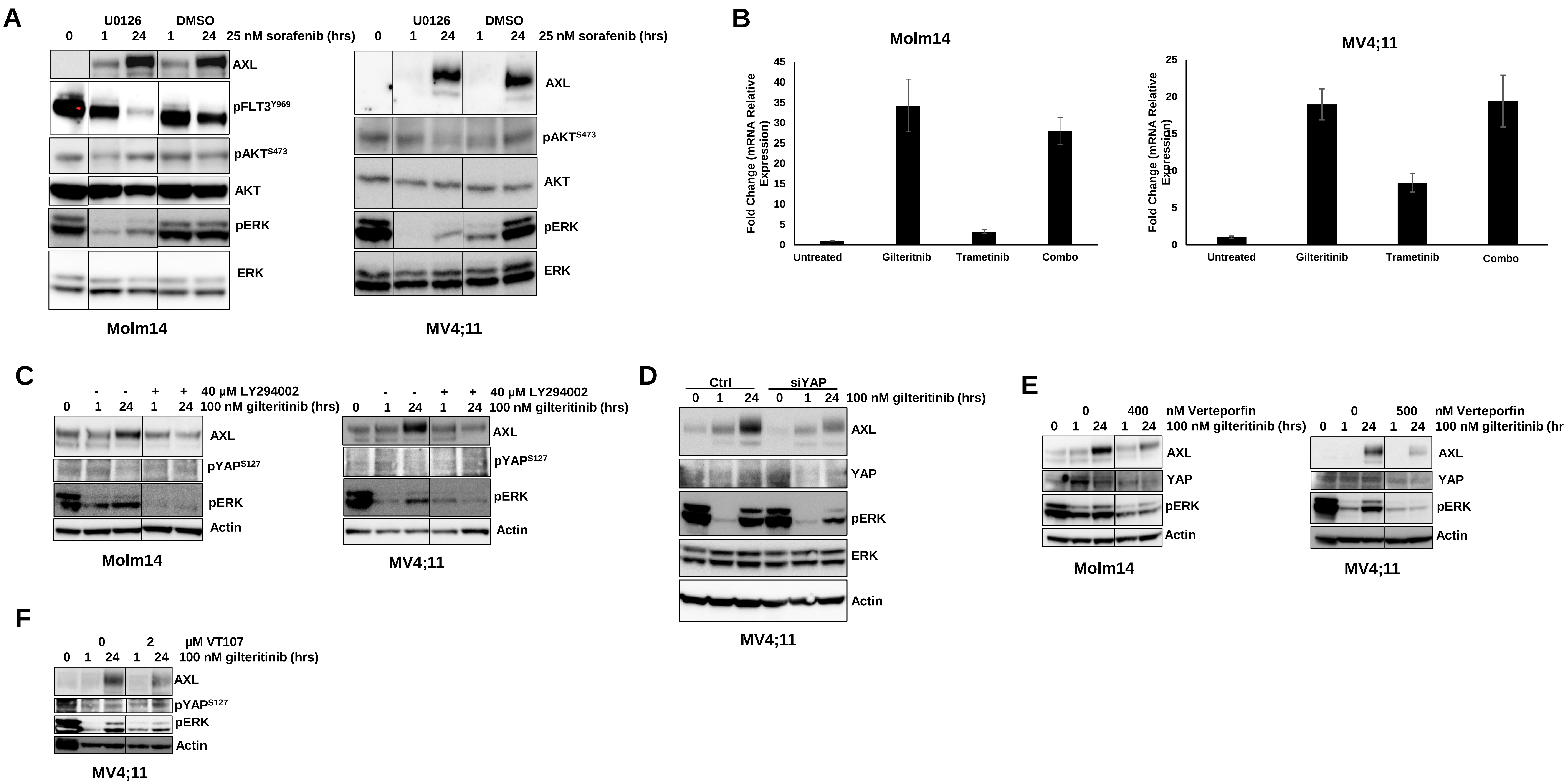

Supplementary Figure 4

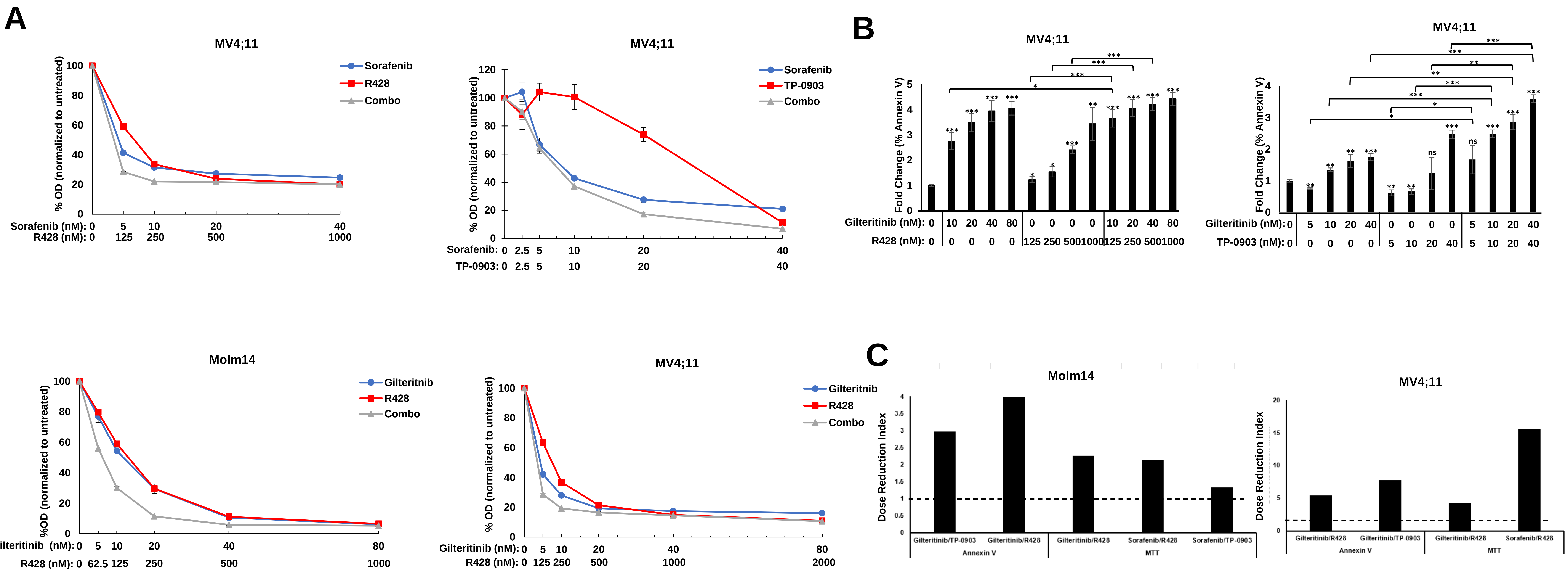

Supplementary Figure 5

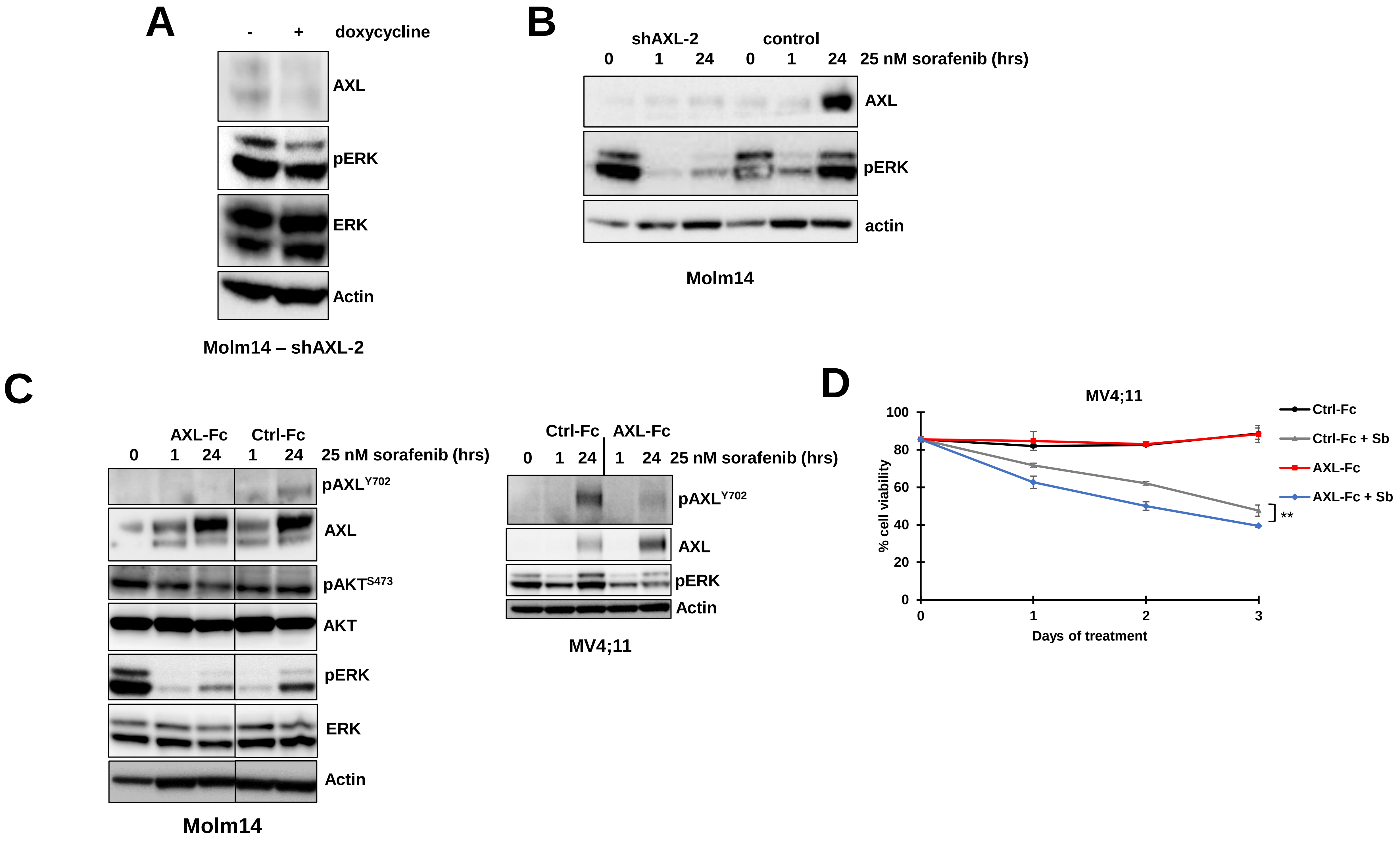

Supplementary Figure 6

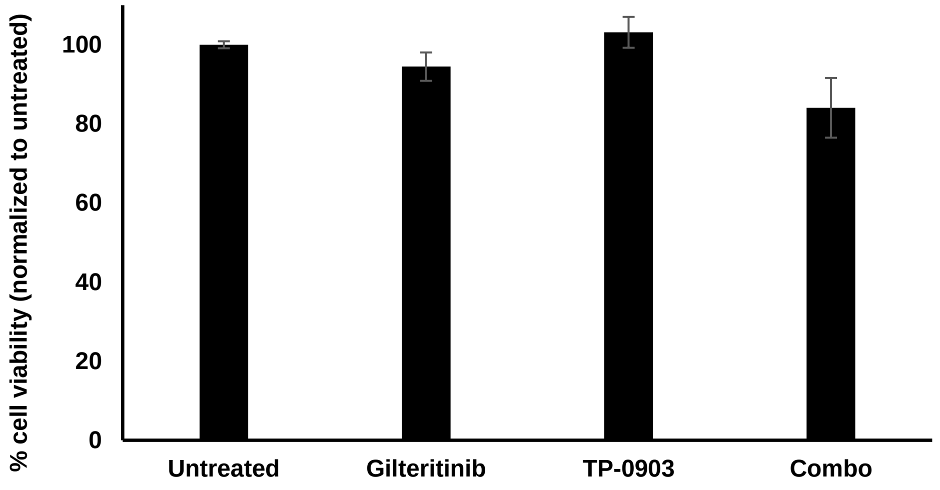

148
Newly Diagnosed

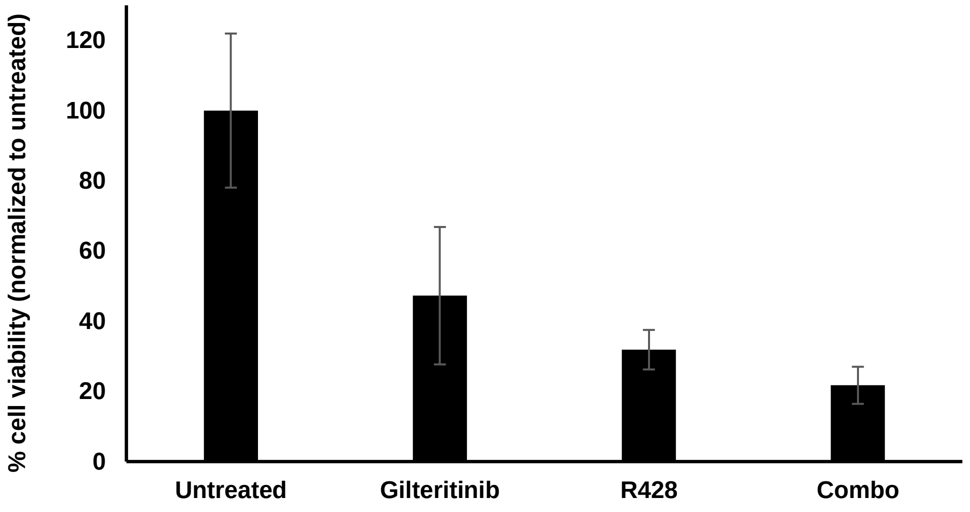

863
Newly Diagnosed

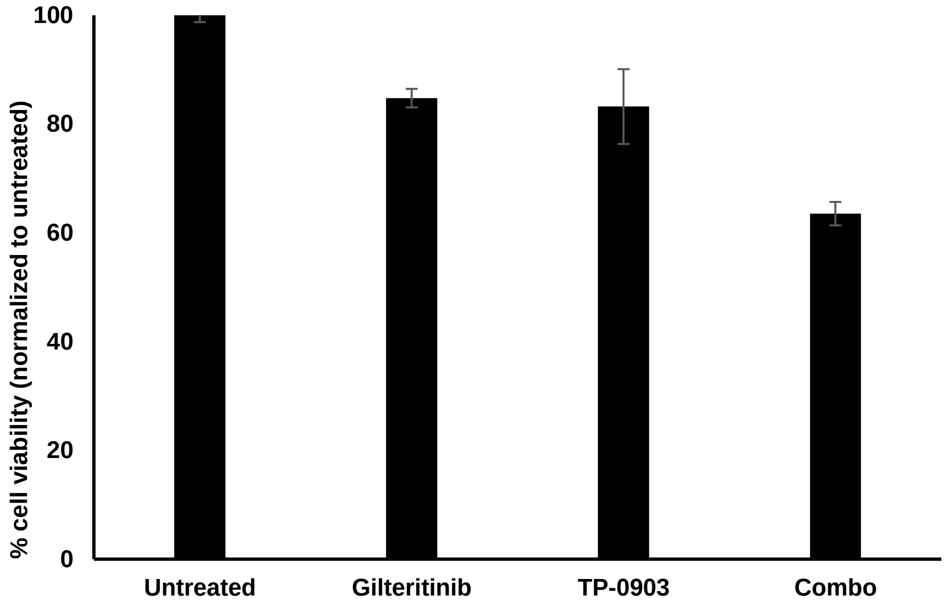

092
Relapsed

Supplementary Figure 7

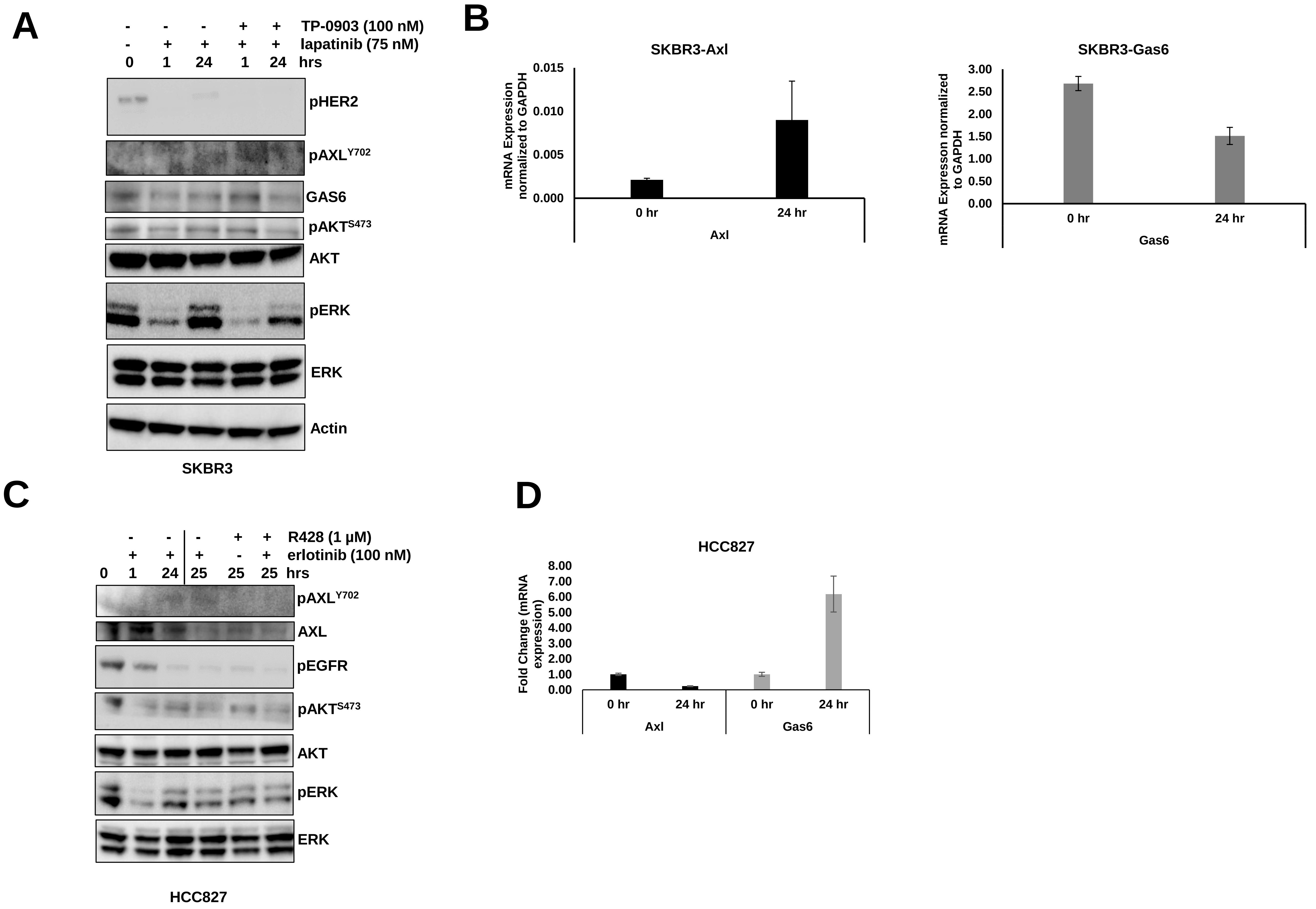

Supplementary Figure 8

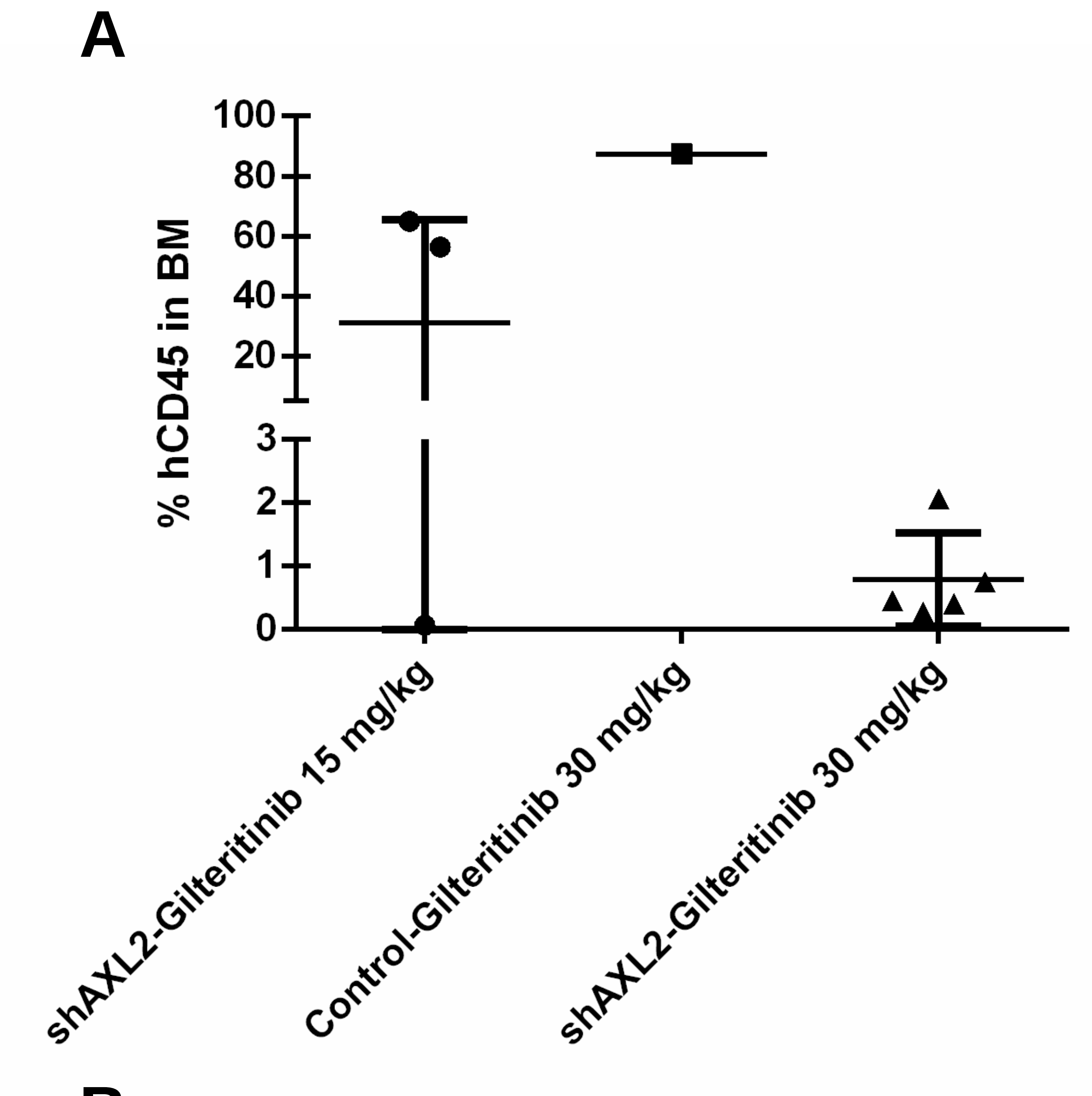

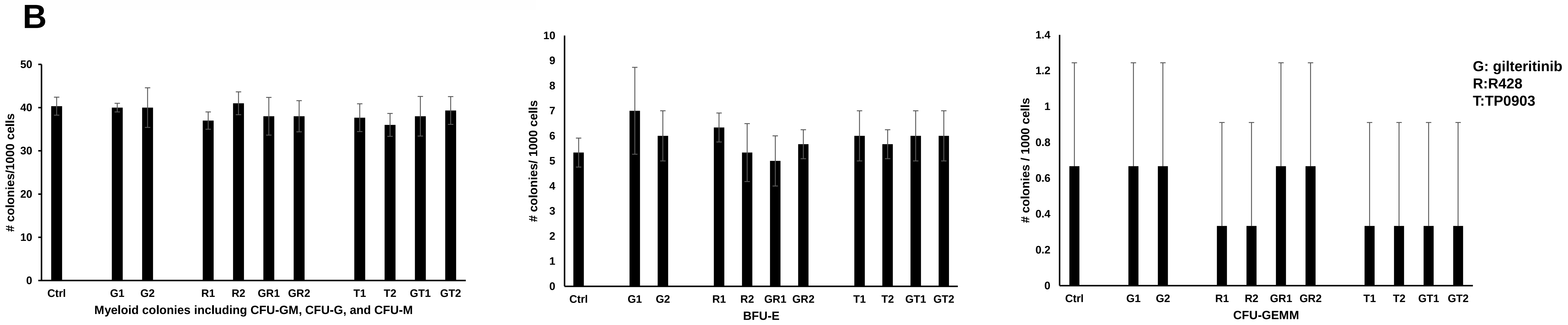

Supplement: Supplementary file 1 — Fig. S1. FLT3 TKI treatment increases AXL activation and lowers GAS6 levels in FLT3/ITD cell lines. Fig. S2. AXL overexpression increases ERK phosphorylation and proliferation. Fig. S3. AXL levels are unaffected by MEK inhibition but are reduced by PI3K and YAP inhibition. Fig. S4. Combination FLT3 TKI and AXL inhibitors decrease proliferation and increase apoptosis. Fig. S5. AXL inhibition through genetic knockdown and ligand trapping affects pERK rebound. Fig. S6. AXL and FLT3 inhibition decrease FLT3/ITD primary cell viability. Fig. S7. TKI‐induced AXL perturbations are observed in other RTK‐driven cancers. Fig. S8. Recovery leukemia burden of shAXL knockdown mice. Table S1. Combination Index (CI) values for FLT3 TKI in combination with AXL inhibitors. Table S2. AML patient characteristics. [file MOL2-19-1386-s001.pdf]
